# Genetics in the Ocean's Twilight Zone: Population Structure of the Silvery Lightfish Across Its Distribution Range

**DOI:** 10.1111/eva.70188

**Published:** 2026-03-23

**Authors:** María Quintela, Alejandro Mateos‐Rivera, Roger Lille‐Langøy, François Besnier, Konstantinos Tsagarakis, Naiara Rodríguez‐Ezpeleta, Miguel Bao, Martin Wiech, Lucilla Giulietti, Sonia Rábade‐Uberos, Fran Saborido‐Rey, Malika Chlaida, Espen Strand, Sofie Knutar, Eva García‐Seoane, Webjørn Melle, Bjørn‐Erik Axelsen, Kevin A. Glover

**Affiliations:** ^1^ Population Genetics Group Institute of Marine Research Bergen Norway; ^2^ Hellenic Centre for Marine Research Institute of Marine Biological Resources and Inland Waters Athens Greece; ^3^ AZTI Basque Research and Technology Alliance (BRTA), Marine Research Sukarrieta Spain; ^4^ Seafood Hazards Group Institute of Marine Research Bergen Norway; ^5^ Integrated Marine Ecology Institute of Marine Research (IIM‐CSIC) Vigo Spain; ^6^ National Institute of Fisheries Research (INRH) Casablanca Morocco; ^7^ Plankton Group Institute of Marine Research Bergen Norway; ^8^ Sustainable Oceans and Coasts Møreforsking AS Ålesund Norway

**Keywords:** Atlantic, genetic structure, *Maurolicus muelleri*, Mediterranean, mesopelagic fish, silvery lightfish, SNPs

## Abstract

The large estimates of mesopelagic fish biomass have long fuelled harvesting interests in the relatively untouched twilight zone of the ocean. The silvery lightfish—one of the most abundant species inhabiting the North Atlantic mesopelagic layer—is a candidate for such a fishery despite its enormous ecological importance and the insufficient knowledge about its population genetic structure. To address this knowledge gap, 863 individuals sampled across the North Atlantic Ocean and into the Mediterranean Sea were genotyped using a panel of 170 genome‐wide SNP loci. Analyses revealed habitat‐driven differentiation into three main units: Mediterranean Sea, oceanic samples, and Norwegian fjords. These groups were not completely isolated from each other as introgression from the Mediterranean Sea was detected in the Eastern Atlantic façade extending from Moroccan waters northward to 47° N, within an otherwise genetically homogeneous oceanic cluster. The complex topography of the Greek Seas seemed to shape the genetic structure in the Mediterranean Sea whereas along the Norwegian coastline, sills did not appear to hinder genetic exchange among fjords ranging 200 km apart, likely reflecting a combination of the position of the species in the water column and its swimming ability. This genetic information should be integrated with ecological and demographic properties to outline the management boundaries of this species prior to any eventual fishery attempt.

## 1 Introduction

The mesopelagic zone, located between 200 and 1000 m depth, and hosting up to 90% of the total oceanic fish biomass (Irigoien et al. 2014), is a largely unexplored area. The limited amount of sunlight at these depths explains the synonym ocean's twilight zone, driving unique adaptations such as bioluminescence and diel vertical migration in the organisms inhabiting it. The enormous biomass of mesopelagic fish, with estimates ranging between 1.8 and 19.5 Gt (Hidalgo and Browman 2019; Irigoien et al. 2014; Proud et al. 2018), fueled interests for the exploitation of a resource intended to aid in meeting the demands of the expanding world population, both in terms of human food security and animal feed in a time when over a third of the worldwide fisheries operate beyond their biologically sustainable levels (FAO 2020; Froese et al. 2025). However, sustainable exploitation of the mesopelagic resources encounters challenges such as the limited knowledge about trophic interactions, life histories, behaviour, biomass, diversity, nutritional composition, and population genetic structure of the mesopelagic organisms.

Mesopelagic organisms play a key ecological role in the Biological Carbon Pump (BCP), which is the suite of processes that collectively account for the ocean's biologically driven sequestration of carbon from the atmosphere and land runoff to the ocean interior and seafloor sediments (Boyd et al. 2019; Ducklow 2001; Sigman and Haug 2006). Photosynthesis by phytoplankton in the sunlit surface ocean involves net uptake of CO_2_ from the atmosphere, whereas the transfer of carbon from the surface to the deep ocean, amounting to 1300 Pg C yr^−1^, occurs via distinct pathways including gravitational settling of organic particles, mixing and advection of suspended organic carbon, as well as active transport by vertically migrating metazoans (Nowicki et al. 2022). The diel vertical migrations (DVM) of many mesopelagic fish species that feed on zooplankton near the surface at night and return to deep layers during the day actively export carbon from the surface to deeper water masses and contribute to the biological pump (Hidaka et al. 2001; Robinson et al. 2010; Shreeve et al. 2009) in what is regarded as the “largest daily migration of animals on earth” (Hays 2003). The active transport mediated by diel vertical migration sequesters more carbon than the physical pump (subduction + vertical mixing of particles), that is, 1.0 vs. 0.8 Pg C because of deeper remineralization depths (Stukel et al. 2023). Without it, the levels of atmospheric CO_2_ would be about 400 ppm higher than at present (Boyd 2015; Sanders et al. 2014).

The silvery lightfish, *Maurolicus muelleri* (Gmelin, 1789) is a small but abundant mesopelagic fish belonging to the family Sternoptychidae (see Grimaldo et al. (2020)). The genus *Maurolicus* was initially regarded as monotypic with a single species, *M. muelleri* (Gmelin, 1789), of cosmopolitan distribution. Later on, following the identification of differences in combinations of morphometric characters, the genus was split into 15 species (Rees et al. 2020, Figure 1). However, many of them display overlapping ranges of meristic and morphometric traits and thus remain poorly characterized (Rees et al. 2017). Mitochondrial (16S rRNA and COI) and nuclear (ITS‐2) gene sequences revealed groups conflicting with previously recognized species: (1) a ‘Northern’ clade comprising *M. muelleri* and *M. amethystinopunctatus* , (2) a ‘Southern’ clade comprising *M. australis* , *M. walvisensis* (also *M. japonicus* ) and (3) an Eastern Equatorial and Western North Atlantic *M. weitzmani* (Rees et al. 2017). Synonymization is proposed for *M. muelleri* and *M. amethystinopunctatus* , with limited morphological variation likely to reflect physical and biological differences experienced North/South of the sub‐polar front (Rees et al. 2017). In a follow‐up study involving multiple locations worldwide, *M. muelleri* and *M. australis* were regarded as potentially a single species, but since no shared haplotypes were found between the two disjunct groups, they were kept separately (Rees et al. 2020). *M. muelleri* is thus distributed in the North Atlantic Ocean and Mediterranean Sea.

**FIGURE 1:**
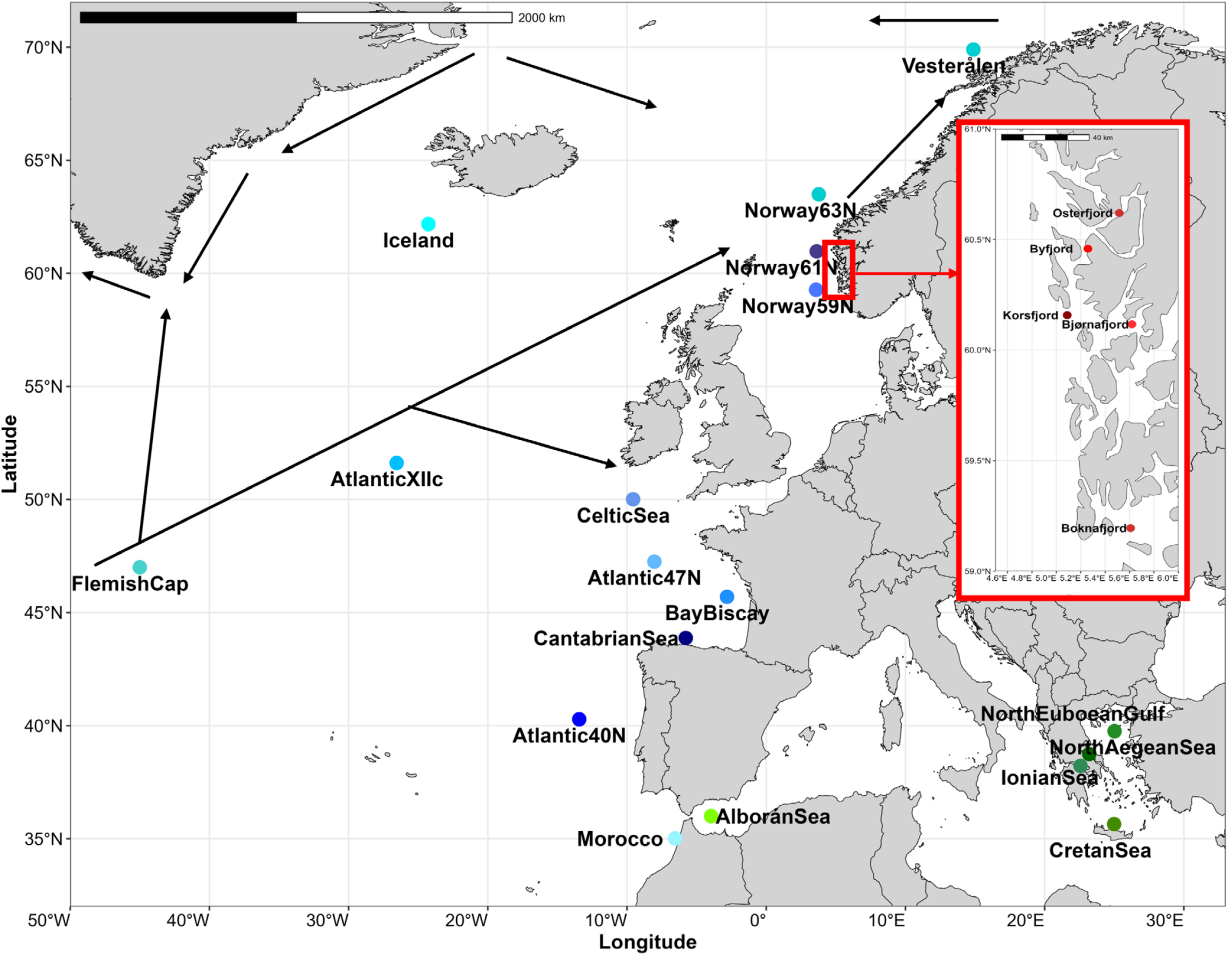
*Maurolicus muelleri* samples. Blue dots depict the Ocean samples whereas green dots depict the Mediterranean ones. The fjord samples, zoomed in the box to the right, are signalled with red dots. Arrows depict the direction of the main ocean currents. For three of the sampling sites (i.e., Iceland, Cantabrian Sea and Bay of Biscay) consisting of different nearby stations, the geographic coordinates correspond to the most central site. The map was created using the geom_sf() function in the R package *ggplot2* (Wickham 2016).

*Maurolicus muelleri* is one of those species conducting DVM that play a critical role in marine ecosystems both as part of the BCP as well as linking primary consumers and higher trophic levels such as seabirds, mammals, cephalopods and larger fish (e.g., Cherel et al. 2010; Drazen and Sutton 2017), including highly valuable commercial species. The species forms distinct sound scattering layers at depth (SLs) and can be acoustically detected even from the youngest ontogenetic stages due to their prominent swim bladder. In the Norwegian fjords, the SL composed of adults displays DVM between April and June (Goodson et al. 1995; Rasmussen and Giske 1994), whereas in early winter (January) adult fish remain in deeper waters while juveniles perform DVM as well as nocturnal midnight sinking after dusk (Baliño 1993; Giske et al. 1990; Goodson et al. 1995). Fully grown individuals can achieve swimming speeds of 2–7 body lengths per second (i.e., 8–30 cm·s^−1^) (Torgersen and Kaartvedt 2001).

In addition to taxonomic considerations, the literature dealing with population structure in *M. muelleri* is sparse and limited to few genetic studies. In western Norway, allozymes revealed that samples collected from five fjords displayed varying degrees of genetic differentiation to each other, and to a sample collected in the North Sea (Suneetha and Nævdal 2001). In the Bay of Biscay, thousands of SNPs obtained from RADseq revealed no clear evidence of population structure as well as lower observed heterozygosity than anticipated (Rodríguez‐Ezpeleta et al. 2017). Finally, mitochondrial DNA genes (COI, 12S rRNA, and 16S rRNA) revealed a lack of genetic structure among samples taken from the Greek Seas (Sarropoulou et al. 2022).

The outline of biologically correct management units or stocks is one of the multiple requirements of sustainable fisheries and intends to prevent the overexploitation of unique spawning components (e.g., see Kerr et al. (2017) for review). As this ecologically crucial species is a potential candidate for exploitation in a commercial fishery, there is a need to fill the knowledge gap regarding its ocean‐wide genetic structure. In addition, when outlining management areas, special attention needs to be directed to identify locally adapted populations or subdivided ones as they might have different sustainable yield levels and be more prone to the negative effects of overfishing (Waples et al. 2008; Pinsky and Palumbi 2014). Furthermore, the range shifts that many marine species are already experiencing in the face of climate change should also be taken into consideration (Dahms and Killen 2023; Palacios‐Abrantes et al. 2022).

The aim of the present study was to provide the first ocean‐wide examination of the genetic structure of *M. muelleri* . Several hundred individuals sampled across most of the species' distribution range including both sides of the Atlantic Ocean (between longitudes 26° W–25° E and latitudes 35°–70° N) were high‐throughput genotyped using a suite of 170 SNP loci. Using ecological parallelism with the mesopelagic glacier lanternfish *Benthosema glaciale* (Quintela et al. 2024), we hypothesize that the genetic patterns of differentiation of *M. muelleri* could be habitat‐related with three major units corresponding to Norwegian fjords, the open Atlantic Ocean and the Mediterranean Sea, respectively.

## 2 Materials and Methods

### 2.1 Sampling

In the period 2017–2024, 941 individuals were collected in 23 sampling sites ranging across a large part of the North Atlantic distribution of the species (Figure 1). Based upon our previous experience with another widely distributed mesopelagic fish, *Benthosema glaciale* (Quintela et al. 2024), three main habitats were aimed at: Norwegian fjords (i.e., five samples around 60° N), Mediterranean Sea (i.e., one sample from the Alborán Sea plus four Eastern samples from the Greek Seas), and open sites in the Atlantic Ocean (i.e., thirteen samples within latitudes 35°–70° N and longitudes 26° W°–25° E). Wherever available, 50 individuals were collected per location. Two of the Greek samples came from the enclosed, deep and isolated gulfs of Corinth (Ionian Sea) and of North Euboea, with geophysical features similar to the Norwegian fjords (Kapelonis et al. 2023). Fin clips were taken and stored in ethanol 96% prior to DNA isolation, which was conducted using SPRI paramagnetic beads from the Beckman Coulter DNAdvance kit (A48706). DNA concentration was quantified using the Thermo Scientific NanoDrop 8000.

### 2.2 SNP Development

Four sampling sites belonging to the different habitats were selected for SNP mining following the procedure used in Quintela et al. (2024): Mediterranean Sea (Ionian Sea), fjords (Boknafjord) and oceanic (Atlantic_47N and Celtic Sea) both to capture as much genetic variability as possible as well as to reduce ascertainment bias in the resulting SNP panel. From these samples, RNA‐free DNA was extracted using the Beckman Coulter DNAdvance kit (A48706). DNA integrity was assessed by agarose gel electrophoresis and DNA concentration was quantified using Thermo Fisher Qubit dsDNA Broad Range (Q32853). Equimolar amounts of DNA from 10 individuals per site were pooled, and one library was prepared per pool using Illumina DNA prep. Pooled samples were then sequenced on a NovaSeq X using 1/8 of a NovaSeq X Series 25B flow cell (150 PE). The package *fastp* v.1.0 (Chen et al. 2018) was used for data preprocessing, which included deduplication, adaptor removal and analysis of over‐represented sequences. FASTQC v.0.11.5 (https://www.bioinformatics.babraham.ac.uk/projects/fastqc/) and MULTIQC v.1.7 (https://github.com/MultiQC/MultiQC) were used to output graphics and statistics of the data quality control.

The lack of a published genome assembly led us to develop a draft assembly on our own sequencing data using MEGAHIT v.1.2.9 (Li et al. 2015, 2016) testing Kmer from 41 to 73. Scaffolding was performed with SoapDeNovo v.2 (https://github.com/aquaskyline/SOAPdenovo2) using 63 as Kmer value. The obtained draft assembly consisted mostly of small contigs, shorter than 500 bp. However, 2156 contigs were larger than 10 kb and represented together 27 Mb. The four paired‐end pooled samples were then mapped against this 27 Mb short genome using BWA v.0.7.17 (Li and Durbin 2010) with default parameters for BWA aln and BWA sampe. Variant sites were called with the mpileup function (Li 2011) from samtools v.1.9 (Li et al. 2009). The R package *vcfR* v.1.15.0 (Knaus and Grünwald 2016, 2017) was used to visualise the distribution of coverage and mapping quality across all variant sites. Only Single Nucleotide Polymorphic (SNP) variants were retained if they fulfilled the following criteria: phred‐scaled quality score (QUAL > 500), coverage larger than 120× and lower than 300× for each pooled sample site, and both alternative and reference allele represented in at least two samples out of four. No attempt whatsoever was directed at producing diagnostic SNPs, that is, at retaining SNPs that maximized the differentiation (*F*_ST_) between habitats or sites within habitats (in the case of the Atlantic). To test for any potential bias induced by the filtering procedure, the percentage of polymorphic loci per site using unfiltered and filtered SNPs was assessed in the four locations used for SNP development. Finally, SNPs located less than 200 bases from another polymorphic site (SNP or indel) were removed to keep only SNPs with stable primer sequences. The goal was to genotype as many individuals as possible across the species' distribution range using a high‐throughput SNP genotyping approach in a reasonably affordable manner. Therefore, the Assay Designer option of the Typer v.5 program (Agena Biosciences CA, USA) was used to create multiplex reactions containing a maximum of 25 SNPs each without making any attempt to enhance the diagnostic ability of any of those multiplexes.

### 2.3 SNP Genotyping

The number of multiplexes selected for throughput SNP genotyping was based upon a trade‐off among the number of loci per multiplex, the multiplex confidence score and the economic resources available. Therefore, eight multiplexes containing 200 SNPs in total were finally retained to genotype the full set of samples. Primers were designed, and 941 individuals were genotyped using the Sequenom MassARRAY iPLEX Platform as described by Gabriel et al. (2009). From the Flemish Cap (NW Atlantic Ocean), only 10 individuals were obtained, which were sampled both for fin clips and muscle. Thus, DNA was extracted from both tissues and individuals were amplified twice to conduct positive controls and reproducibility tests.

### 2.4 Genetic Structure

To assess if the set of individually genotyped SNPs would accurately discriminate between individuals in a population, the genotype accumulation curve was built using the function *genotype_curve* in the R (Team 2025) package *poppr* v.2.0.0 (Kamvar et al. 2014) by randomly sampling × loci without replacement and counting the number of observed multilocus genotypes (MLGs). This was repeated r times for 1 locus up to n‐1 loci, creating n‐1 distributions of observed MLGs. The observed (*H*_*o*_) and unbiased expected heterozygosity (u*H*_e_) as well as the inbreeding coefficient (*F*_IS_) were computed for each sample with GenAlEx v.6.1 (Peakall and Smouse 2006). Likewise, the genotype frequency of each locus and its direction (heterozygote deficit or excess) were compared with Hardy–Weinberg expectations (HWE) using the program GENEPOP v.4.0.6 (Rousset 2008) as was linkage disequilibrium (LD) between pairwise loci. The False Discovery Rate (FDR) correction of Benjamini and Hochberg (1995) was applied to *p*‐values to control for Type I errors. Non‐parametric Kruskal–Wallis's rank sum test followed by post hoc Dunn's test was applied to perform a comparison of estimates of genetic diversity among habitats. Data conversion into the appropriate formats was conducted using PGDSpider v.2.1.1.5 (Lischer and Excoffier 2012).

Unsupervised analysis of genetic structure was conducted with Principal Component Analysis (PCA) using the function *dudi.pca* in the *ade4* v.1.7‐23 package (Dray and Dufour 2007) in R (Team 2025) after replacing missing data with the mean allele frequencies, using no scaled allele frequencies (scale = FALSE). In addition, the Bayesian clustering approach implemented in STRUCTURE v.2.3.4 (Pritchard et al. 2000), and conducted using the software ParallelStructure (Besnier and Glover 2013), was used to identify genetic groups under a model assuming admixture and correlated allele frequencies without using LOCPRIORS. Ten runs with a burn‐in period consisting of 100,000 replications and a run length of 1,000,000 MCMC iterations were performed for *K* = 1 to *K* = 10 clusters. To determine the number of genetic groups, STRUCTURE output was analysed using two approaches: (a) the *ad hoc* summary statistic Δ*K* of Evanno et al. (2005), and (b) the Puechmaille (2016) four statistics (MedMedK, MedMeanK, MaxMedK and MaxMeanK), both implemented in StructureSelector (Li and Liu 2018). Finally, the ten runs for the selected Ks were averaged with CLUMPP v.1.1.1 (Jakobsson and Rosenberg 2007) using the FullSearch algorithm and the G' pairwise matrix similarity statistic, and graphically displayed using bar plots.

Supervised genetic structure using geographically explicit samples was assessed using the Analysis of Molecular Variance (AMOVA) and pairwise *F*_ST_ (Weir and Cockerham 1984), both computed with Arlequin v.3.5.1.2 (Excoffier et al. 2005). Furthermore, the relationship among samples was examined using the Discriminant Analysis of Principal Components (DAPC) (Jombart et al. 2010) implemented in the R (Team 2025) package *adegenet* v.2.1.11 (Jombart 2008) in which groups were defined using geographically explicit locations. To avoid overfitting, both the optimal number of principal components and discriminant functions to be retained were determined using the cross‐validation function (Jombart and Collins 2015; Miller et al. 2020).

The relationship between genetic (*F*_ST_) and geographic distance was examined to investigate if it adhered to the expectations of an “Isolation by Distance” pattern (IBD), that is, increasing genetic differentiation with geographic distance as a result of restricted gene flow and drift (Rousset 1997; Slatkin 1993; Wright 1943). A two‐tailed Mantel (1967) test was conducted using PASSaGE v.2 (Rosenberg and Anderson 2011) and significance was assessed via 10,000 permutations. The matrix of pairwise shortest geographic distance by water (i.e., avoiding land masses) was calculated with the R (Team 2025) package *marmap* v.1.0.12 (Pante and Simon‐Bouhet 2013). Data on summer temperature measured at 200 m depth using a grid of ¼ ° and averaged for the period 2005–2012 was retrieved from the NOAA database (National Oceanic and Atmospheric Administration).

Putative clines of allele frequency extending from the easternmost Atlantic coast into the Mediterranean Sea were investigated via the latitudinal sliding‐window approach developed by Pereira et al. (2018) using the R scripts provided by the authors. In a second step, loci identified as displaying clines were subjected to a geographic cline analysis conducted using the R package HZAR v.0.2‐5 (Derryberry et al. 2014) over a circa 8000 km transect starting in Vesterålen and finishing in the Cretan Sea. The 15 models implemented in HZAR were fitted to the allele frequency of the candidate loci to determine the position, width and shape of the cline over the total geographic distance. The reference cline was built using STRUCTURE Q‐score and, in both cases, the best cline model was decided upon AIC scores.

## 3 Results

### 3.1 SNP Development and Genotyping

Pool genome sequencing generated a total of 216,150 non‐filtered SNPs, revealing clear genetic differentiation among the three putative habitats as hypothesized. The first principal component (PC1), explaining 43.5% of the variation (Figure S1a), separated the Mediterranean Sea (Ionian Sea) while PC2 (29.7%) further differentiated the remaining samples. After filtering based on phred‐scaled quality score, coverage, and representation of both alleles in at least two of the four samples, 12,000 SNPs were retained. Patterns of habitat differentiation in this filtered dataset closely mirrored those from the unfiltered data, with PC1 and PC2 explaining 39.45% and 31.23% of the variation, respectively (Figure S1b). In both datasets, the strongest differentiation (largest *F*_ST_) was observed between the Ionian Sea and Boknafjord (Table S2). Notably, when comparing the Mediterranean and the oceanic samples, the Atlantic_47N sample was genetically closer to the Mediterranean Sea than the Celtic Sea sample.

Finally, the 2034 SNPs that fulfilled the criteria of being ≥ 200 bp apart from another polymorphic site were arranged into multiplex reactions. Eight of those multiplexes, containing a total of 200 SNPs, were retained to genotype the full set of 941 individuals.

The proportion of polymorphic loci per sample increased across the filtering process from 43% to 51% in the unfiltered dataset to 85% (Ionian Sea) and 98% (Atlantic samples) in the individually genotyped SNPs (Figure S2). Fjord and oceanic samples exhibited a consistent increase across filtering steps, whereas the Ionian Sea sample showed minor fluctuations.

### 3.2 Genetic Structure

Statistical analyses were restricted to the subset of 170 well‐functioning polymorphic loci obtained out of the 200 screened ones (Table S1 compiles the reasons for discarding the 30 ones). Even though the threshold of acceptance of missing data per individual was 20%, only 3 of the 863 finally retained individuals showed the maximum missing data allowed, whereas 63% of them displayed ≤ 5%. These 863 individuals were distributed in 23 samples across three different habitats: fjords, Atlantic Ocean and Mediterranean Sea.

The 10 fish collected in the Flemish Cap and used for quality control and reproducibility revealed that DNA revealed similar performance regardless of being extracted from muscle or finclips, except in one of the individuals where finclips failed in 92% of the 200 tested loci. In the remaining nine pairs and excluding the differences due to the failure of amplification in one of the individuals, 14 discrepancies (i.e., homozygote vs. heterozygote) were found (0.77%).

The number of markers needed to confidently differentiate individuals in a sample set can be graphically evaluated using the genotype accumulation curve (GAC). When the curve reaches a plateau, adding more loci no longer significantly increases the number of unique genotypes detected. In this case, the plateau of the GAC was reached with 17 out of the 170 retained loci, indicating that approximately 10% of the SNPs displayed enough power to discriminate between unique genotypes from the same sample (Figure S3). The Kruskal‐Wallis test detected significant differences in genetic diversity across habitats (*p* of 0.005 and 0.002, respectively for *H*_O_ and u*H*_e_, and *p* = 0.05 for the percentage of polymorphic markers) with lower values reported for the Mediterranean Sea in all cases (Table 1). Dunn's a posteriori test revealed significant differences for the three estimates between Mediterranean Sea and oceanic habitats and between fjords and Mediterranean Sea for *H*_O_. Out of the 330,395 performed tests for LD, 3% were significant after FDR. Deviations from HWE were found in 49 out of the 3910 loci by population tests after FDR (1.25%).

**TABLE 1:** *Maurolicus muelleri* : Sample summary statistics obtained for the set of 170 SNP loci. Sampling sites are arranged into habitats with geographic coordinates in decimal degrees; number of individuals (N); percentage of polymorphic loci (Pol. loci); observed heterozygosity, *H*_o_ (mean ± SE); unbiased expected heterozygosity, u*H*_e_ (mean ± SE); inbreeding coefficient, *F*_IS_ (mean ± SE); number of deviations from Hardy–Weinberg equilibrium (Dev. HWE) and number of deviations from Linkage Disequilibrium (Dev. LD) at *α* = 0.05 both before and after False Discovery Rate (FDR) correction. As some of the sampling sites consist of different nearby stations, the geographic coordinates indicated per sample are an average of all trawls for simplicity.

Habitat	Sample	Latitude	Longitude	*N*	Pol. loci	*H*_o_	u*H*_e_	*F*_IS_	Dev. HWE (FDR)	Dev. LD (FDR)
Fjords	Osterfjord	60.62	5.52	45	93.5	0.304 ± 0.013	0.314 ± 0.012	0.014 ± 0.015	17 (2)	504 (441)
	Byfjord	60.46	5.26	39	91.2	0.297 ± 0.013	0.305 ± 0.013	0.005 ± 0.014	9 (1)	461 (411)
	Korsfjord	60.16	5.09	44	92.4	0.308 ± 0.013	0.318 ± 0.013	0.017 ± 0.014	10 (2)	485 (428)
	Bjørnafjord	60.12	5.62	49	97.1	0.311 ± 0.013	0.320 ± 0.012	0.013 ± 0.013	10 (4)	558 (493)
	Boknafjord	59.20	5.61	46	90.6	0.311 ± 0.014	0.310 ± 0.013	−0.009 ± 0.014	9 (1)	485 (422)
Oceanic	Vesterålen	69.90	14.88	15	89.4	0.301 ± 0.014	0.325 ± 0.013	0.039 ± 0.022	8 (2)	320 (275)
	Norway 63° N	63.50	3.78	18	85.9	0.250 ± 0.013	0.306 ± 0.014	0.122 ± 0.023	17 (2)	246 (211)
	Iceland	62.18	−24.27	10	87.1	0.296 ± 0.017	0.315 ± 0.014	0.007 ± 0.026	7 (0)	136 (95)
	Norway 60° N	60.98	3.63	50	94.1	0.309 ± 0.013	0.322 ± 0.013	0.025 ± 0.013	12 (2)	557 (506)
	Norway 59° N	59.27	3.58	46	95.3	0.318 ± 0.014	0.319 ± 0.013	−0.007 ± 0.014	16 (1)	524 (462)
	AtlanticXIIc	51.62	−26.55	47	92.4	0.311 ± 0.014	0.318 ± 0.013	0.026 ± 0.016	18 (5)	565 (500)
	Celtic Sea	50.01	−9.56	38	97.6	0.332 ± 0.013	0.332 ± 0.011	−0.004 ± 0.016	16 (3)	627 (555)
	Flemish Cap	47.00	−45.00	10	82.4	0.295 ± 0.017	0.309 ± 0.015	−0.018 ± 0.027	6 (0)	221 (174)
	Atlantic 47° N	47.26	−8.03	41	98.2	0.352 ± 0.011	0.363 ± 0.010	0.016 ± 0.015	13 (2)	670 (593)
	Bay of Biscay	45.70	−2.82	28	97.1	0.350 ± 0.012	0.358 ± 0.011	0.009 ± 0.016	8 (1)	654 (582)
	Cantabrian Sea	43.88	−5.78	31	98.2	0.344 ± 0.013	0.351 ± 0.011	0.001 ± 0.018	16 (2)	584 (526)
	Atlantic 40° N	40.28	−13.43	49	99.4	0.345 ± 0.011	0.362 ± 0.010	0.028 ± 0.013	14 (1)	804 (714)
	Morocco	35.01	−6.54	49	98.8	0.334 ± 0.012	0.343 ± 0.011	0.016 ± 0.014	13 (2)	699 (639)
Mediterranean	Alborán Sea	36.00	−3.96	15	82.4	0.243 ± 0.015	0.261 ± 0.014	0.025 ± 0.022	10 (2)	175 (146)
	N Aegean Sea	39.76	25.02	53	82.4	0.244 ± 0.015	0.255 ± 0.014	0.037 ± 0.016	20 (5)	685 (609)
	N Euboean Gulf	38.75	23.20	46	95.3	0.295 ± 0.013	0.308 ± 0.013	0.028 ± 0.015	14 (3)	582 (517)
	Ionian Sea	38.22	22.57	47	84.7	0.258 ± 0.014	0.269 ± 0.014	0.041 ± 0.015	15 (4)	449 (394)
	Cretan Sea	35.65	24.99	47	82.9	0.250 ± 0.015	0.252 ± 0.014	−0.006 ± 0.013	7 (2)	348 (305)

**FIGURE 2:**
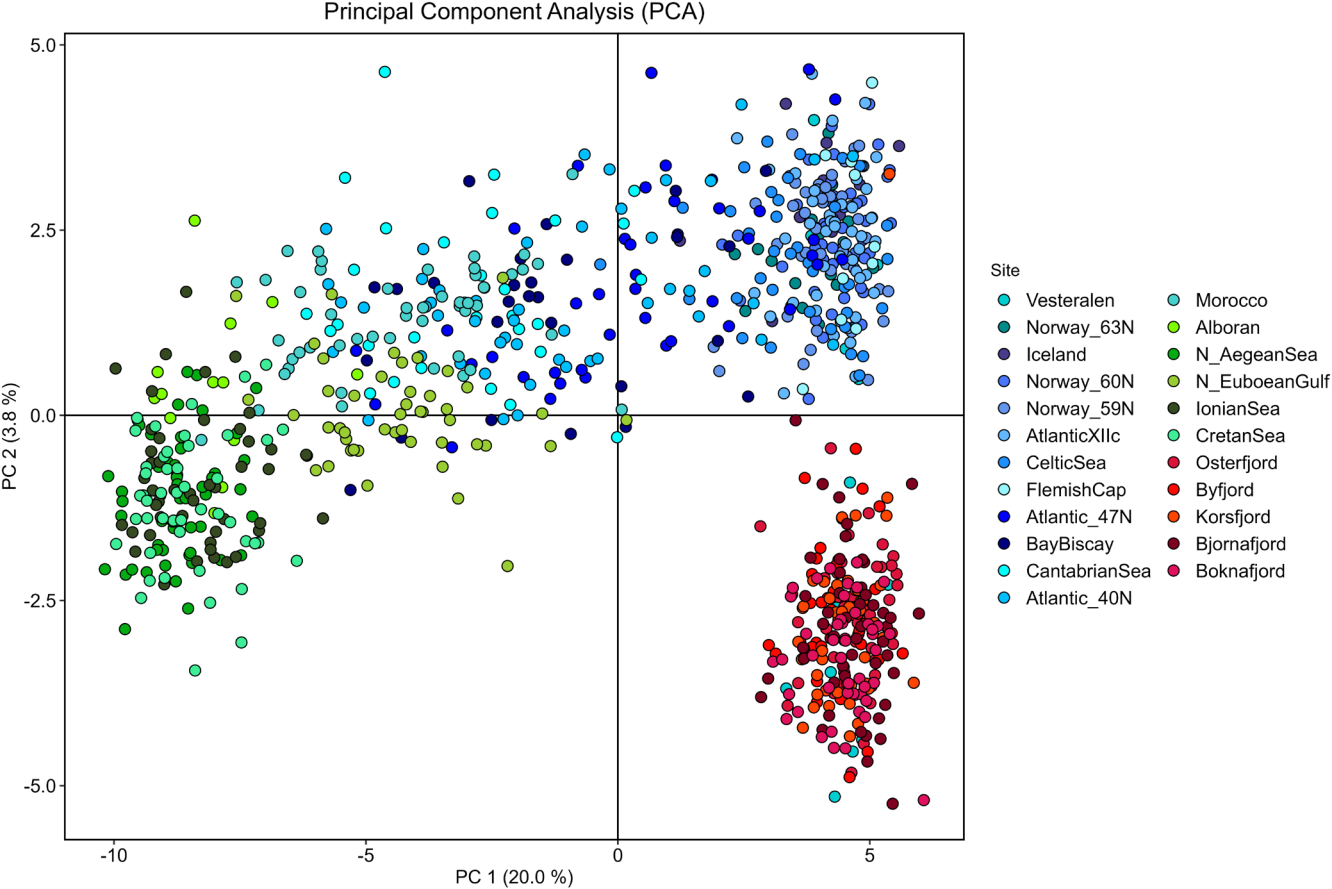
Principal Component Analysis (PCA) of *Maurolicus muelleri* genotyped at 170 loci. Blue dots depict the Ocean samples; red dots indicate fjord individuals and green dots represent the Mediterranean ones.

**FIGURE 3:**
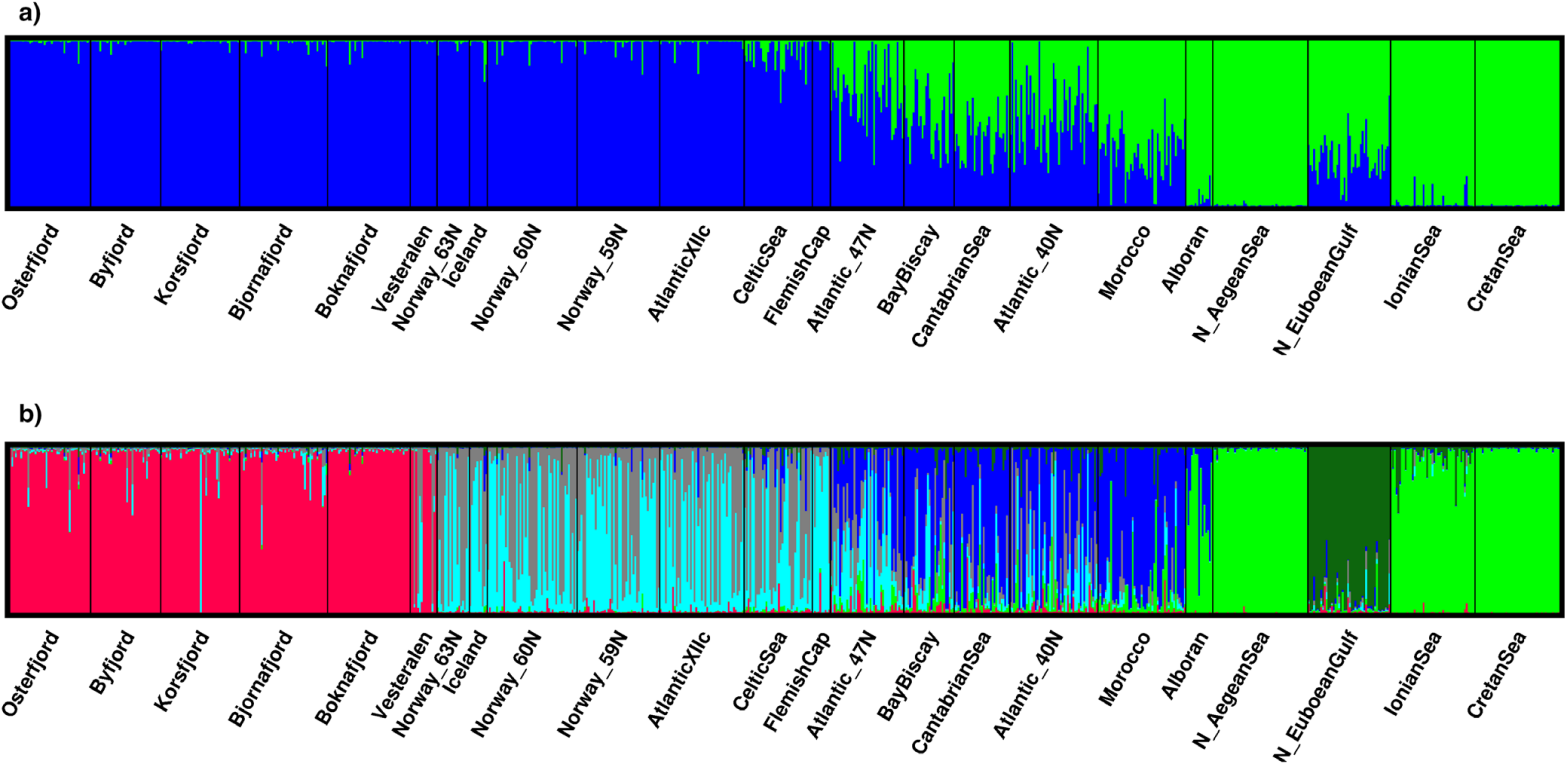
Barplot representing the proportion of *Maurolicus muelleri* individuals' ancestry to cluster at (a) K2 and (b) K7 after Bayesian clustering in STRUCTURE as determined by the a posteriori analyses conducted using Evanno's test and Puechmaille's statistics, respectively.

The first axis of the PCA biplot, accounting for 20% of the variation, neatly separated the Mediterranean Sea from the fjords and part of the oceanic individuals. However, a transition could be observed between the N. Euboean Gulf and some of the Atlantic samples such as Morocco, the Cantabrian Sea or the Bay of Biscay (Figure 2). The second axis (3.8%) separated the fjords and the oceanic samples. In STRUCTURE a posteriori analyses, the Evanno test strongly suggested *K* = 2 as the most likely number of genetic groups (Δ*K* = 33,400, Figure S4). This first division singled out the Mediterranean Sea samples while revealing a decreasing cline of the Mediterranean genetic profile into the oceanic samples from Morocco northwards to the Bay of Biscay (Figure 3a). The uniformity of the Mediterranean Sea samples was disrupted in the Greek sample of the N. Euboean Gulf. At *K* = 3 and *K* = 4, fjord and oceanic clusters became evident while the cline Mediterranean–Atlantic was still present in the southern Atlantic samples (Figure S5a,b). The Mediterranean Sea sample from N. Euboean Gulf revealed a different cluster at *K* = 5 (Figure S5c). Puechmaille's solution of *K* = 6 (Figure 3b) reflected the genetic uniformity of the fjord samples, the distinctness and rather uniform Mediterranean genetic profile (excluding the N. Euboean Gulf) as well as two admixed clusters in the Atlantic Ocean (a northern one from Norway 63° N to Flemish Cap and a southernmost one ranging from 47° N to Morocco). Interestingly, from *K* = 3 onwards, the sample from Vesterålen deviated from the oceanic profile and displayed a seemingly physical mixture of fish belonging to the fjord (majority) and northern Atlantic clusters.

**FIGURE 4:**
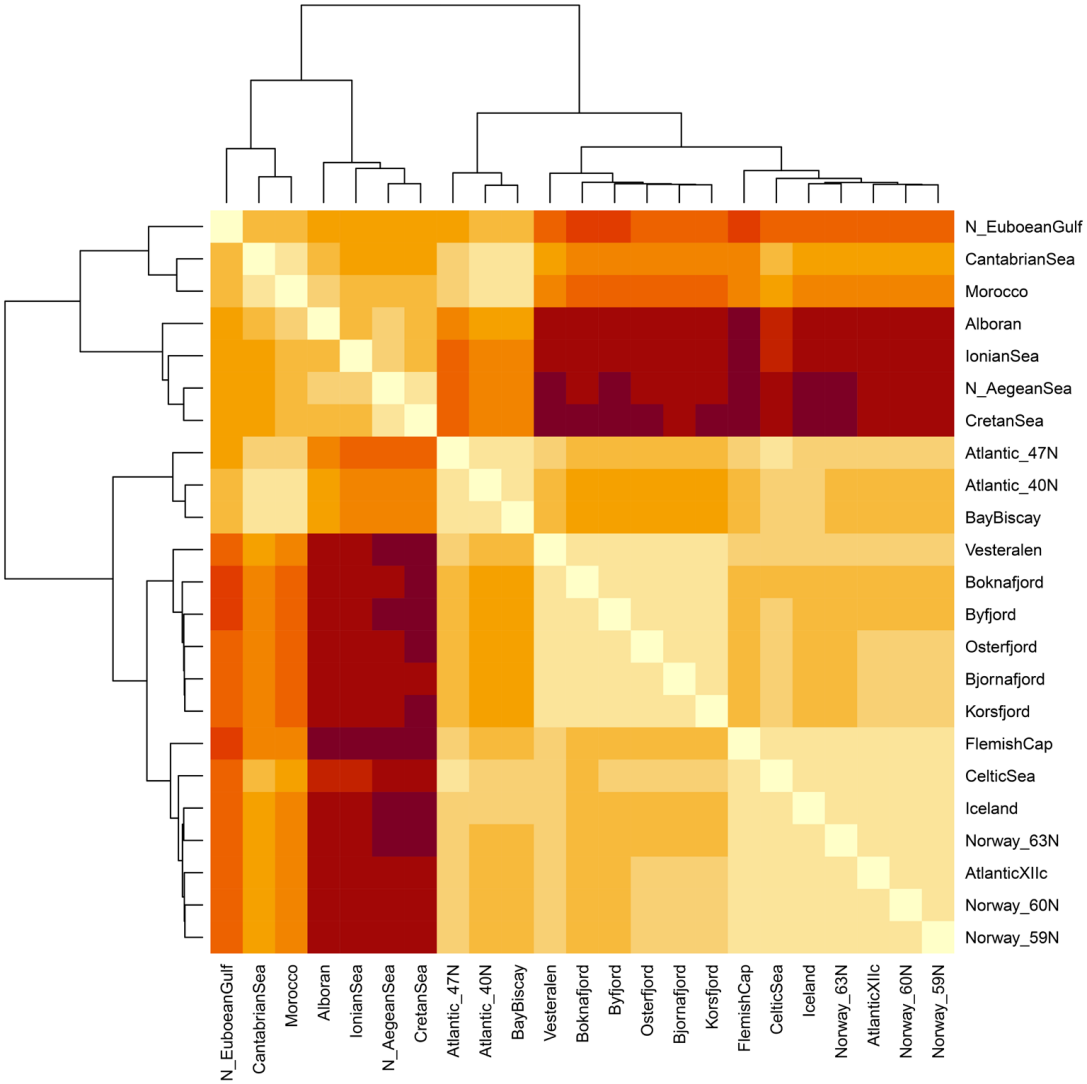
*Maurolicus muelleri* : Heatmap *F*_ST_ coupled with dendrogram. Pairwise values and significance can be found in Table S3. Colours depict the degree of differentiation: Beige colours indicating lower differentiation and moving towards dark brown to indicate larger differentiation.

High levels of genetic differentiation were detected on a per‐locus basis, with 150 out of 170 loci significantly different from zero after FDR, and 83 of them with *F*_ST_ values ranging between 0.1 and 0.8. Hierarchical AMOVA revealed significant structure both among habitats (*F*_CT_ = 0.158, *p* < 0.001), among samples within habitats (*F*_SC_ = 0.057, *p* < 0.001) and within samples (*F*_ST_ = 0.206, *p* < 0.001) with 15.8% of the variation hosted among habitats and 79.4% within samples. The dendrogram coupled with the pairwise *F*_ST_ (Figure 4) revealed a first dichotomic division separating the Mediterranean Sea, Morocco and the Cantabrian Sea from all the remaining sites. Interestingly, not only did the sample from the N. Euboean Gulf cluster with the latter Atlantic samples, but it also showed lower genetic differentiation towards both the Atlantic Ocean and the fjords than any of the Mediterranean Sea samples (Table S3). In the second branch of the tree, the fjords formed their own ramification while joining to Vesterålen whereas the Ocean samples were divided into the northernmost plus westernmost ones versus the ones between 47° and 40° N. Most of the pairwise *F*_ST_ values were significantly different from zero (Table S3) and strong differentiation was found among habitats, particularly towards the Mediterranean Sea but exceptions were detected within habitats. Within fjords, the only significant differentiation was found between the two farthest most sites, that is, Osterfjord and Boknafjord separated by 200 km (*F*_ST_ = 0.007, *p* = 0.001). In the Mediterranean Sea, the only samples that did not differ from each other were the N. Aegean Sea and the Cretan Sea. The complicated topography of the Greek Seas with very confined body waters such as in the N. Euboean Gulf and the Gulf of Corinth (Ionian Sea) seems to hamper the genetic exchange whereas the 462 km of distance between the N. Aegean Sea and the Cretan Sea sampling sites did not seem to be a limitation for gene flow. Within the oceanic samples, 71% of the pairwise comparisons were significant. However, almost no differentiation was detected between the samples ranging from Vesterålen southwards to the Celtic Sea together with the three westernmost ones (Iceland, AtlanticXIIc and FlemishCap). In the group of oceanic samples ranging between Morocco northwards to 47° N, both the Cantabrian Sea and Morocco were significantly different from the remaining (pairwise *F*_ST_ ranging between 0.005 and 0.025) whereas no differentiation was detected among Atlantic_40° N, Atlantic_47° N and the Bay of Biscay.

**FIGURE 5:**
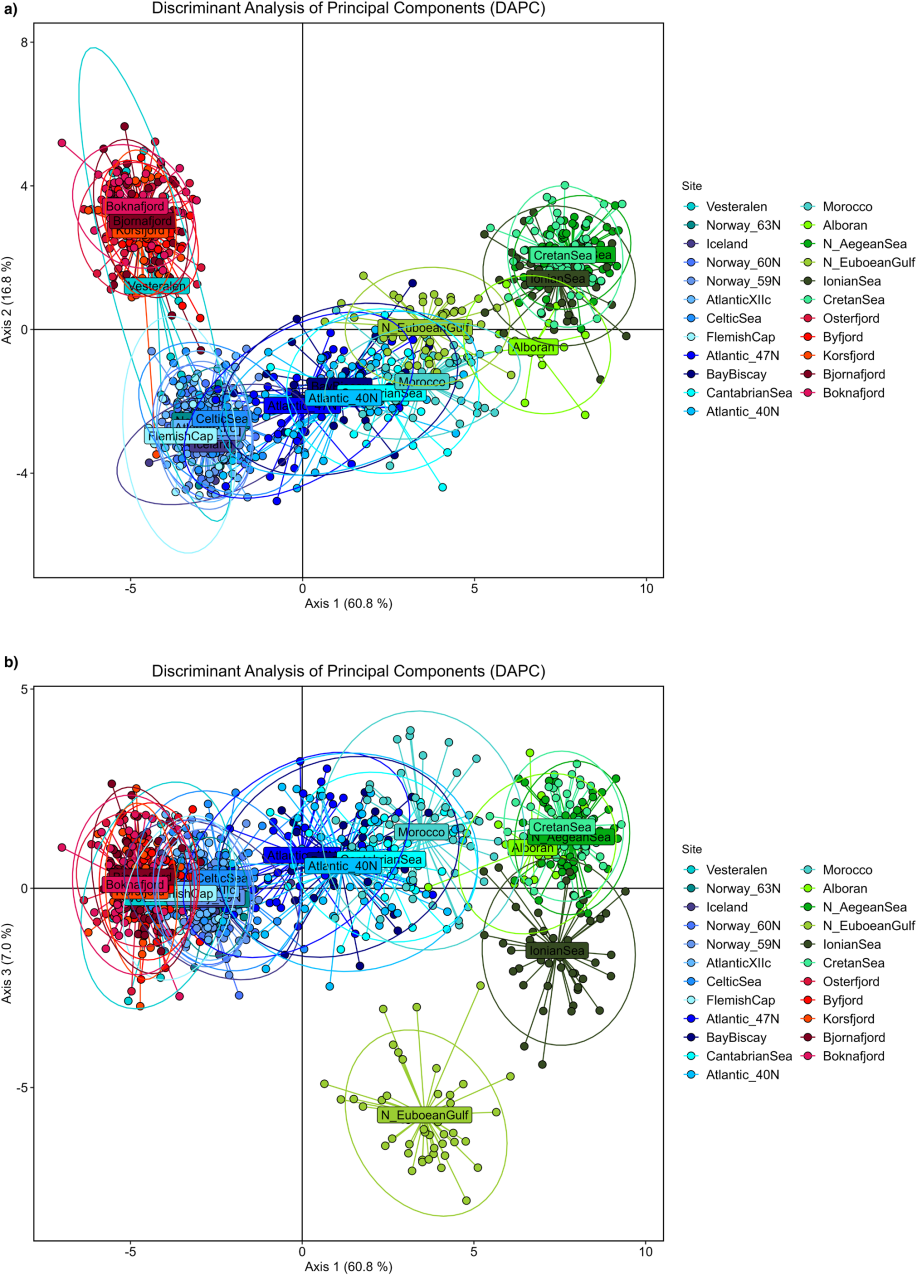
Genetic differentiation among *Maurolicus muelleri* samples assessed with 170 SNP loci using Discriminant Analysis of Principal Components (DAPC) after retaining 100 principal components and 3 discriminant functions: (a) axis 1 and 2, and (b) axis 1 and 3. Individuals from different sampling sites are represented by coloured dots.

**FIGURE 6:**
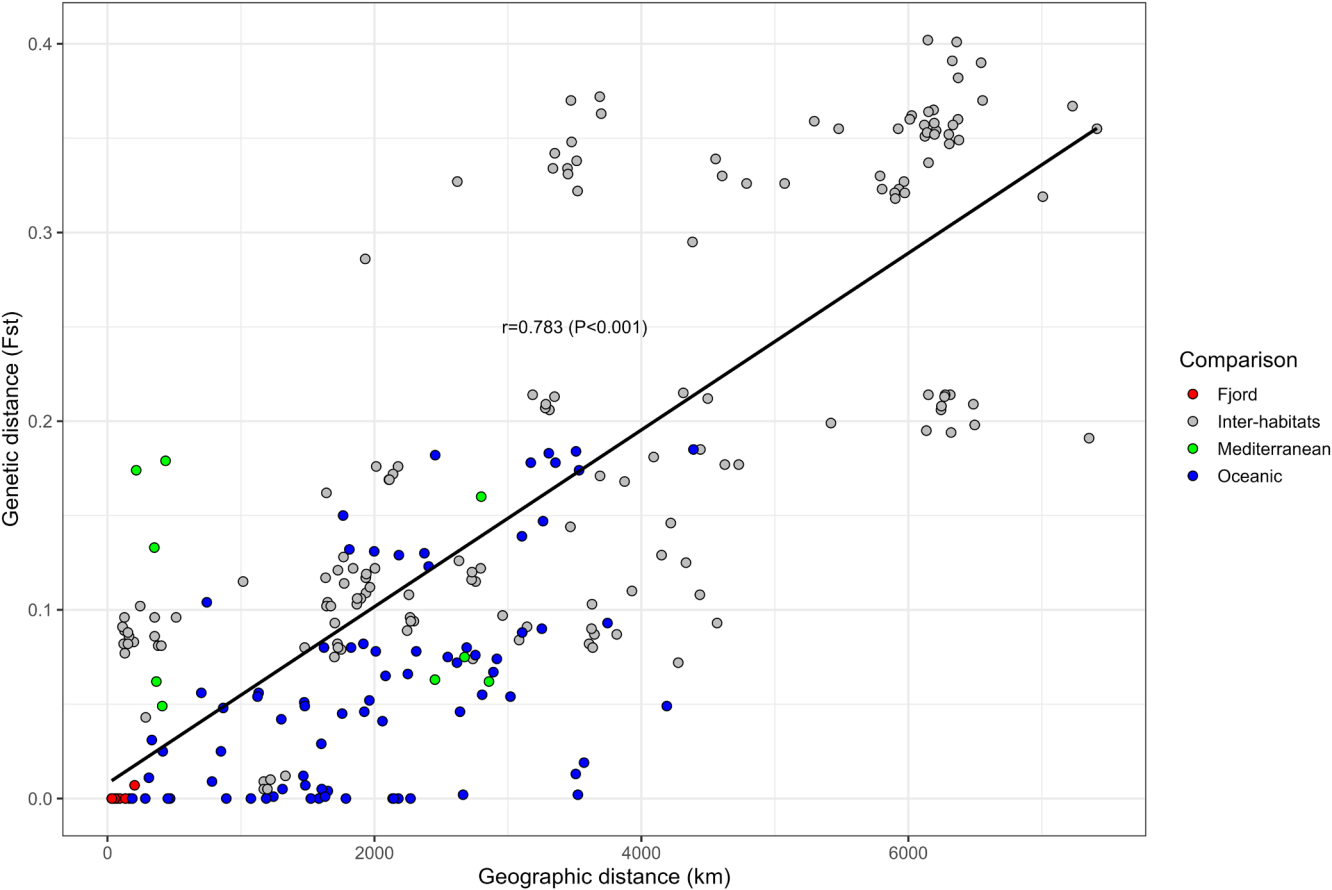
*Maurolicus muelleri* : Mantel test showing significant correlation between shortest geographic distance by water (km) and genetic distance (*F*_ST_). Colours depict the samples involved in the comparisons within (red, blue, green) and across habitats (grey).

The DAPC revealed both differences among the three habitats (ocean, fjords and Mediterranean) as well as a certain continuity among them. Thus, the first axis of differentiation (60.8% of the variation) showed the uniqueness of the fjord individuals as well as a continuum between the Atlantic samples and the Mediterranean Sea, together with the total lack of overlapping between the Mediterranean Sea and the Northernmost and Westernmost Atlantic ones (Figure 5a). The second axis of differentiation (16.8%) further singled out the fjord individuals. As formerly seen, some of the individuals from Vesterålen clustered with the fjord samples whereas the remaining clustered with the Northern group of the Atlantic. The third axis of differentiation (7%) singled out the sample from the N. Euboean Gulf (Figure 5b). The positive and strong correlation (*p* = 0.783, *p* < 0.001) between the shortest water distance between samples and the genetic distance measured as pairwise *F*_ST_ was indicative of a pattern of Isolation by Distance (Figure 6).

**FIGURE 7:**
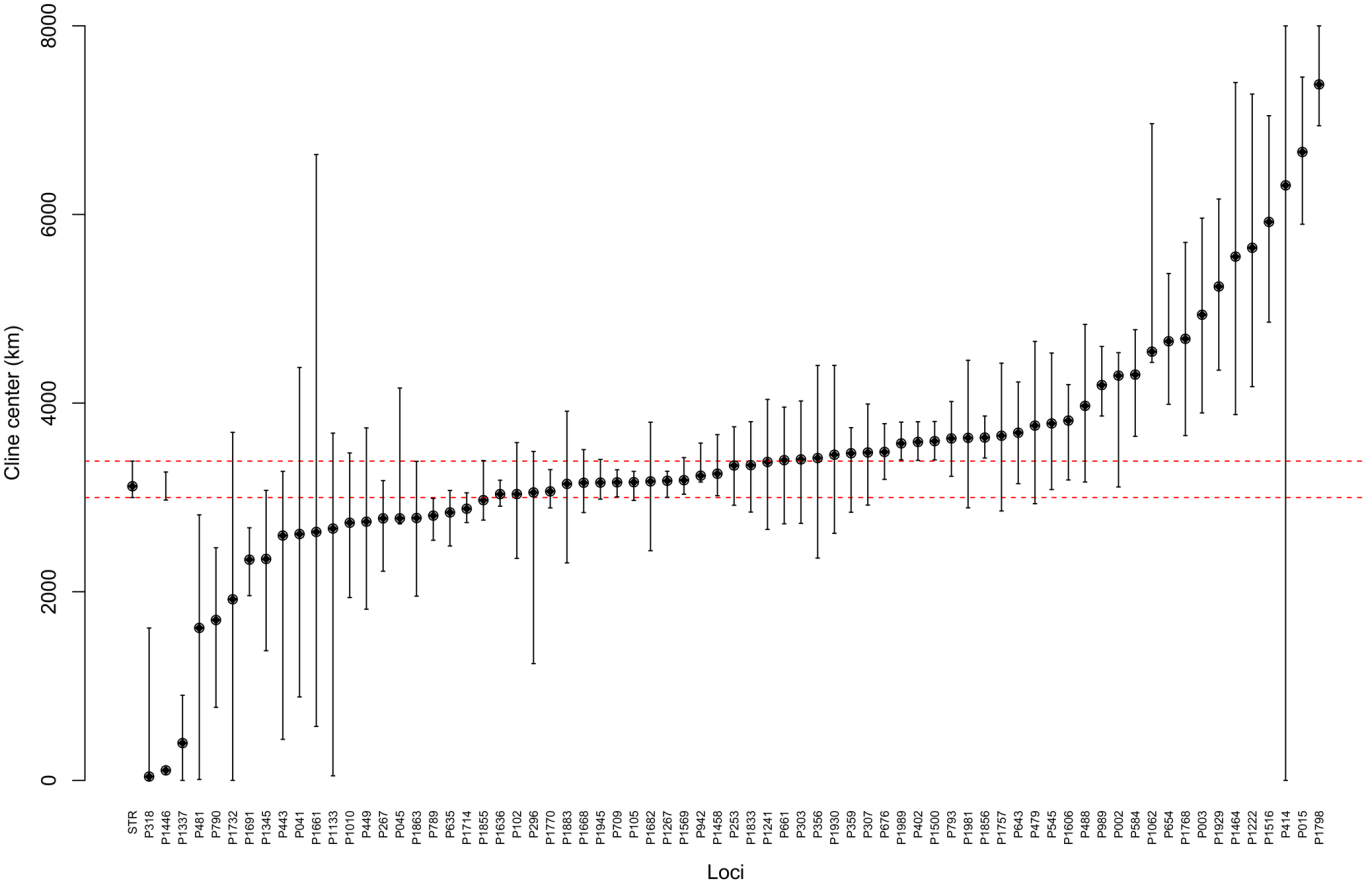
*Maurolicus muelleri* : Summary of HZAR analyses: Cline centre and width for the loci showing signs of latitudinal clines. Red dotted lines represent the limits of the reference cline built with STRUCTURE Q‐score.

The allele frequencies of 72 out of the 170 loci showed signs of a latitudinal cline and only in one of them, the difference between maximum and minimum frequencies was < 0.2 (in 49 of the loci, delta frequency ranged between 0.5 and 1). STRUCTURE Q‐score also adhered to a clinal pattern with delta frequency of 0.99 (see Figure S6). HZAR analyses revealed that the cline was centred within the limits of the reference cline—that is, around 3000–3400 km south from Vesterålen (Figure 7)—for 18 of the 72 loci, which corresponds to a latitude around 44°–47° N (Table S4).

## 4 Discussion

This study addresses a key knowledge gap in the population genetic structure of a prospective fisheries resource: the silvery lightfish, *Maurolicus muelleri* , across much of its distribution range. The main outcome is the finding of three main genetic groups, inhabiting the Mediterranean Sea, the Norwegian fjords and the Atlantic Ocean, each exhibiting different levels of intragroup variation.

### 4.1 SNP Development

The genetic markers used in the current study stem from a two‐step procedure: first, mining SNPs from pool sequencing data, and second, genotyping a subset of non‐diagnostic loci in nearly one thousand individuals using a high‐throughput SNP genotyping method. Ascertainment bias was attenuated by including individuals from the three distinct habitats—open sites in the Atlantic Ocean, Mediterranean Sea and Norwegian fjords—in the pool sequencing step, thereby maximizing the capture of existing genetic variation. To evaluate the potential bias introduced during filtering, the percentage of polymorphic loci per site was compared between unfiltered and filtered datasets. Polymorphism increased steadily across all four sites except the Ionian Sea, where a slightly fluctuating pattern was observed, potentially indicating an underestimation of the genetic differentiation between the Mediterranean Sea and the remaining locations. Nevertheless, strong differentiation was consistently detected among habitats and within them, particularly in the Mediterranean Sea and Atlantic Ocean. Interestingly, despite the geographic proximity (~330 km) between the Atlantic_47N and Celtic Sea samples, pairwise *F*_ST_ when comparing them with the Ionian Sea was remarkably lower for Atlantic_47N both using filtered and unfiltered SNP. This pattern could be interpreted as a hint of the genetic introgression of the Mediterranean into the eastern façade of the Atlantic Ocean, a signal later confirmed when individually genotyping all individuals.

The methodological approach used in this study was recently used to mine 121 SNPs displaying remarkable differentiation across habitats in the glacier lanternfish *Benthosema glaciale* (Quintela et al. 2024). Likewise, 91 SNPs mined from ddRAD sequencing unravelled patterns of differentiation in European sprat (Quintela et al. 2020) later confirmed by whole genome sequencing (Pettersson et al. 2024). The success of former experiences, the similarity between the patterns of differentiation revealed by pool genome sequencing and the 170 genotyped SNPs, in addition to the outcome of the Genotype Accumulation Curve (Figure S3), made us confidently rely on the discriminating capacity of the SNP array used here.

### 4.2 Habitat Differentiation

The hypothesis of habitat‐driven differentiation formulated for *M. muelleri* could not be rejected following the identification of three significantly differentiated genetic groups inhabiting the Mediterranean Sea, the Norwegian fjords and the Atlantic Ocean, respectively. The species contributes to the body of literature on genetically identified ecotypic differentiation detected in marine taxa and driven by environmental factors such as bathymetry as in tusk ( *Brosme brosme* ) (Knutsen et al. 2009) or beaked redfish ( *Sebastes mentella* ) (Benestan et al. 2021), salinity as in European sprat ( *Sprattus sprattus* ) (Pettersson et al. 2024; Quintela et al. 2020), or the distinction between marine vs. coastal habitat as demonstrated in European anchovy ( *Engraulis encrasicolus* ) (Le Moan et al. 2016), long‐snouted seahorse (Hippocampus *guttulatus*) (Meyer et al. 2024; Riquet et al. 2019), northern shrimp ( *Pandalus borealis* ) (Hansen et al. 2021) or Atlantic cod ( *Gadus morhua* ) (Knutsen et al. 2018).

The dispersion of marine taxa between the Atlantic and Mediterranean basins was facilitated by the opening of the Strait of Gibraltar some 5.33 million years ago, although in multiple marine species, the observed genetic patterns of differentiation suggest a limitation to gene flow thus preventing population admixture (see Patarnello et al. 2007 for revision). Thus, in *M. muelleri* , secondary contact could be happening in the western Mediterranean Sea as the Alborán sample seems to be the only sample showing introgression from the Atlantic cluster (Figure 3b). Water exchange between basins occurs through the surface Atlantic inflow and the deeper and saltier Mediterranean outflow; a range of salinities that the euryhaline *M. muelleri* can tolerate. Adults typically remain below the Camarinal Sill during the day (mean depth ~350 m (Olivar et al. 2022)), but they might cross it into the Atlantic Ocean during their diel vertical migration (figure 5 in Sánchez‐Garrido and Nadal (2022)). Larvae, which inhabit upper waters (Olivar et al. 2018), can be transported from the Atlantic Ocean into the Mediterranean Sea by surface currents; however, the gene flow from the Atlantic cluster seems to be largely interrupted beyond eastern Alborán Sea. We therefore hypothesize that *M. muelleri* eggs and larvae enter the Mediterranean Sea via the surface Atlantic inflow, but their survival might be limited by the strong hydrodynamics of the Alborán Sea (Olivar et al. 2022), thus explaining the lack of an Atlantic genetic signal farther east.

Oceanographic features like current patterns and discontinuities shape the genetic connectivity within the Mediterranean Sea (Galarza, Carreras‐Carbonell, et al. 2009; Schunter et al. 2011), the Almería‐Oran Front (AOF) being the major point of genetic break with the Atlantic (Patarnello et al. 2007). Thus, Harmelin and d'Hondt (1993) observed that some Atlantic taxa that can survive in the Alborán Sea are absent from other Mediterranean areas. A combined signature of vicariance, paleoclimate fluctuation and life‐history traits accounts for the Atlantic Ocean–Mediterranean phylogeographical patterns observed in many marine taxa (e.g., Bremer et al. 1996; Nesbø et al. 2000; Patarnello et al. 2007; Aarbakke et al. 2014), with genetic breaks identified in fish (e.g., Bargelloni et al. 2003; Barros‐García et al. 2020; Bremer et al. 1996; Meyer et al. 2024; Riquet et al. 2019; Tine et al. 2014), as well as other taxonomic groups (Fontaine 2016; Jenkins et al. 2019; Lapègue et al. 2023; Pérez‐Losada et al. 2002; Reuschel et al. 2010; Riesgo et al. 2019). The entry of less saline water from the Atlantic through the shallow Strait of Gibraltar represents a barrier to gene flow for multiple species (Galarza, Carreras‐Carbonell, et al. 2009; Galarza, Turner, et al. 2009; Marie et al. 2016) and could account for the much lower mesopelagic fish diversity found in the Mediterranean Sea compared with the adjacent Atlantic waters (Olivar et al. 2022).

Likewise, no genetic structure at mitochondrial cytochrome b was detected in the North Pacific Ocean in the myctophids *Diaphus theta* , *Stenobrachius leucopsarus* , *S. nannochir* displaying different diel vertical migration patterns (Kojima et al. 2009). A lack of microsatellite genetic differentiation coupled with large genetic diversity was also reported for the southern hemisphere myctophid *Electrona antarctica* (de Van Putte et al. 2012). The homogenizing effect of the Southern Coastal Current in the Southern Ocean not only affects this species, but also others such as humped rockcod *Gobionotothen gibberifrons* (Matschiner et al. 2009) and Antarctic silverfish *Pleuragramma antarctica* (Zane et al. 2006) for which gene flow mediated by larval dispersal resulted in weak or absent genetic structure. In *M. muelleri* , almost no differentiation was detected in the Atlantic Ocean in the 3750 km range from Norway 63° N to Flemish Cap. The only northern deviating sample was Vesterålen, which seemed to consist of a physical mixture of genetically distinct fjord and offshore individuals. It could be hypothesized that individuals overcoming the fjord sills could be drifted northwards by the Norwegian Current and retained in the Vesterålen area, eventually in combination with the effect of the intensive upwelling that brings deep‐sea water to Lofoten and Vesterålen (Falk and Nøst 2013). Rodríguez‐Ezpeleta et al. (2017) reported exceptionally low levels of genetic diversity in *M. muelleri* from the Bay of Biscay genotyped at thousands of RAD‐seq SNPs, summoning the Wahlund effect. STRUCTURE barplots in our study suggest that admixture with the Mediterranean could account not only for the substructure proposed by Rodríguez‐Ezpeleta et al. (2017), but would seem to drive the patterns of differentiation in the Atlantic cluster in a similar manner as the introgression with North East Arctic Cod shapes population structure in Norwegian Coastal Cod (Dahle et al. 2018; Jorde et al. 2021).

### 4.3 A Tale of Two Mesopelagic Fishes

The patterns of differentiation of *Maurolicus muelleri* largely resemble those observed in *Benthosema glaciale* in the same geographic area (Quintela et al. 2024), particularly the pronounced divergence among habitats. However, despite their ecological similarities, both species exhibit some striking genetic differences as well, namely a putative chromosome inversion suspected to occur in *B. glaciale* . The arrangement of this putative inversion shows remarkable frequency differences between the Atlantic Ocean and the Mediterranean Sea hindering the recombination after gene flow and thus preventing the development of an admixture gradient in that part of the genome. In consequence, no signature of the Mediterranean Sea genetic profile is detected in the genetically uniform Atlantic Ocean samples. In contrast, *M. muelleri* displays an introgression cline of the Mediterranean cluster into the Eastern Atlantic façade ranging from North Moroccan waters northwards 47° N, which mirrors the introgression between the Atlantic and Mediterranean clades reported for example, in European seabass (Duranton et al. 2018; Robinet et al. 2020). Analogous clinal patterns of differentiation between habitats have been described in the transition between the Baltic Sea and the Atlantic Ocean for many species (reviewed in Johannesson and Andre 2006). However, oceanic samples of *M. muelleri* beyond the area of Mediterranean influence displayed almost no differentiation; that is, from Norway 63° N to Flemish Cap (3570 km distance in a longitude range from 3.78° E to 45° W), which closely aligns with the lack of structure displayed by *B. glaciale* across the Atlantic Ocean over distances up to 3940 km (Quintela et al. 2024). Similarly, the lower genetic diversity detected in the Mediterranean for both species suggests long‐term isolation of this genetic cluster. Differences between the two species are also apparent within the Norwegian fjords. Whereas very little genetic differentiation was detected in *M. muelleri* , consistent with earlier studies using allozymes (Watkins et al. 1996), *B. glaciale* showed significant genetic structure (Quintela et al. 2024). Similarly, Suneetha and Salvanes (2001) found that genetic homogeneity in *B. glaciale* occurred only in fjords with sill depths exceeding 130 m. These sills, which typically form at fjord mouths and restrict deep‐water exchange with adjacent coastal areas, may act as physical barriers limiting gene flow in *B. glaciale* (Kristoffersen and Salvanes 2009); however, such barriers do not appear to influence *M. muelleri* . Differential swimming capacity of both species could be partially responsible for this difference in genetic structure. Tracking of individuals in deep water showed that *B. glaciale* was conspicuously inactive and drifted back and forth with weak tidal currents, essentially acting as plankton while active swimming occasionally occurred in the vertical direction with speeds ranging between < 0.5–1 body length s^−1^ (Kaartvedt et al. 2009). In contrast, *M. muelleri* swimming capacity has been reported to reach speeds of 2–7 body length·s^−1^ (i.e., 8–30 cm·s^−1^) (Torgersen and Kaartvedt 2001). In addition to larger swimming capacity, *M. muelleri* has been reported to stay in upper levels in the water column than *B. glaciale* both during daytime and night time, not only in the Norwegian fjords but also in the Mediterranean Sea and the Atlantic coast (Bañón et al. 2016; Bernal et al. 2023; Giske et al. 1990; Kapelonis et al. 2023; Olivar et al. 2022; Rábade Uberos et al. 2021; Staby et al. 2011) even reaching the surface during night time in the Norwegian fjords (Staby et al. 2011). Furthermore, *M. muelleri* has the capability of changing its behaviour in response to ontogeny and internal state (satiation and hunger) as well as to external stimuli (Staby et al. 2011).

In the Mediterranean, the Ionian and Aegean Seas shape a complex ecosystem of highly irregular coastlines and semi‐isolated deep basins, which in combination with demographic processes as well as hydrological and ecological traits lead to differences in species composition (Kapelonis et al. 2023; Somarakis et al. 2011) as well as to genetic differentiation within species (see Sarropoulou et al. 2022 and references therein). Neither *M. muelleri* nor *B. glaciale* is oblivious to the influence of these factors and therefore the isolation of the Gulf of Corinth in the Ionian Sea—a semi‐closed body of water with limited communication to the open sea via shallow and narrow channels, which shape unique hydrological and topographical traits (Drakopoulos and Lascaratos 1998; Ramfos et al. 2005)—was detected in both species as well as formerly suggested for *B. glaciale* based upon a high number of private mtDNA alleles (Sarropoulou et al. 2022). In contrast, non‐significant differentiation was displayed between the N. Aegean Sea and the Cretan Sea for *M. muelleri* as these were the only samples lacking conspicuous physical barriers to gene flow (see Figure 1).

### 4.4 Life History Traits in *Maurolicus muelleri*

In addition, or maybe because of the reported genetic differentiation across habitats, the life history of *M. muelleri* is known to differ geographically, with maximum age reached in Norwegian waters (Gjøsæter and Kawaguchi 1980). Along the Norwegian coast, the hydrological and topographic characteristics of the fjords may create unique habitat conditions (Farmer and Freeland 1983) and differentiation between offshore and fjords was detected in traits such as sexual dimorphism (Kristoffersen and Salvanes 2001), maturation stage, gonadal investment, relative fecundity and growth rate, which displayed increasing trends from the Norwegian Sea to the coast and towards the fjords (Salvanes and Stockley 1996). The reproductive investment was higher in a fjord with higher predation risk (Bjelland 1995) and fjord fish also matured earlier in the season than offshore (Salvanes and Stockley 1996). The mortality rate was reported higher in offshore areas than within the fjords, which was attributed to fish occupying a depth with higher light levels and therefore more vulnerable to predators (Kristoffersen and Salvanes 1998). Differences in growth, condition and gonad weight indicated different resource levels caused by different population densities (Kristoffersen and Salvanes 1998). However, almost three decades ago, life history differences were suggested to be phenotypic and not genetically driven due to the lack of variation fjord‐offshore displayed by allozymes (Watkins et al. 1996), which contrasts with the significant differentiation detected with SNPs in this study (average *F*_ST_ ≈0.09). Interestingly, in the same paper, Watkins et al. (1996) detected fjord‐offshore differentiation in *B. glaciale* , which was later confirmed by SNPs (Quintela et al. 2024). Fjord vs. offshore differentiation has also been described in other marine taxa with high dispersal capacity such as, for example, tunicate *Ciona intestinalis* (Johannesson et al. 2018), northern shrimp *Pandalus borealis* (Hansen et al. 2021), haddock *Melanogrammus aeglefinus* (Berg et al. 2021), Atlantic cod (Pampoulie et al. 2011; Ruzzante et al. 1997; Westgaard and Fevolden 2007) or European sprat (Pettersson et al. 2024; Quintela et al. 2020).

Genetic differentiation across areas also mirrors the differentiation found in life history traits in *M. muelleri* from the Bay of Biscay, Celtic Sea, and around 59°–63° N in the Norwegian Sea where length‐weight relationships revealed differences associated with the fish's origin, paralleling the annual and daily otolith growth. Likewise, von Bertalanffy growth models parameters increased progressively northwards, in accordance with Bergmann's rule (Alvarez et al. 2024). The authors suggested the existence of separated units, either genetically or morphologically, representing differences in biological parameters as a signal of geographical divergence. Our genetic data confirm that the Bay of Biscay significantly differed from the Celtic Sea as well as from Norwegian coastal samples within the same latitudinal range; likewise, the Celtic Sea differed from one of the Norwegian samples.

Finally, allele frequency clines displayed by 42% of the loci analysed in this study could suggest variations due to environmental factors along the latitudinal gradient. Average temperature correlating with genetic differentiation and producing an Isolation‐by‐Environment (IBE) pattern (Table S5) could be a candidate environmental driver. In other marine taxa, annual variance in sea surface temperature has been shown to produce significant genetic–environment associations (Dorant et al. 2020). However, the significant correlation between geographic distance and temperature found here (*r*_xy_ = 0.79, *p* < 0.001) hampers disentangling the effect of both variables, particularly as Partial Mantel tests correcting IBD by temperature retained significance. Following this line of thought, an interesting feature is the deviating allele frequencies found in the Greek sample from the N. Euboean Gulf, which tended towards the range of values found in the Southeastern Atlantic group in contrast with the remaining Mediterranean Sea samples (Table 2). The N. Euboean Gulf is a ~450 m deep basin that connects with adjacent seas through shallow channels and hosts only two mesopelagic fish species ( *M. muelleri* and *B. glaciale* ). The temperature is some ≈2°C lower than in other Mediterranean Sea regions and it is the only Greek basin that features hypoxic conditions at depths deeper than 350 m (see Figure S7). The lower temperature in combination with the hypoxic conditions and the geographic isolation of the gulf could account for the observed patterns.

**TABLE 2:** *Maurolicus muelleri* : Heatmap of allele frequency per sample for the loci involved in clines that displayed the largest contribution to the DAPC loadings on the first axis. Samples are distributed in blocks corresponding to habitats, that is, fjords, oceanic and Mediterranean, respectively.

Sample	P105	P402	P676	P1636	P1945	P1267	P1458	P1856	P1989	P1446	P1500	P789	P1768	P1691	P307	P1661	P1770	P1062	P790	P303	P1863
Osterfjord	1.000	1.000	1.000	1.000	1.000	0.989	0.989	0.978	0.977	0.956	0.956	0.933	0.909	0.878	0.822	0.789	0.778	0.667	0.644	0.593	0.550
Byfjord	1.000	1.000	1.000	1.000	1.000	1.000	1.000	0.974	0.962	0.974	0.962	0.910	0.864	0.816	0.771	0.857	0.692	0.667	0.647	0.553	0.639
Korsfjord	1.000	1.000	0.989	1.000	1.000	1.000	1.000	0.959	0.983	0.976	0.951	0.929	0.914	0.807	0.725	0.702	0.773	0.684	0.547	0.670	0.538
Bjørnafjord	0.990	1.000	0.990	1.000	1.000	0.969	0.990	0.959	0.990	0.959	0.949	0.929	0.957	0.786	0.745	0.796	0.796	0.717	0.704	0.592	0.543
Boknafjord	1.000	1.000	1.000	1.000	1.000	1.000	1.000	0.957	1.000	0.957	0.946	0.932	0.927	0.772	0.679	0.793	0.826	0.579	0.620	0.611	0.488
Vesterålen	1.000	1.000	1.000	1.000	1.000	1.000	1.000	1.000	1.000	0.955	1.000	0.933	0.864	0.833	0.800	0.900	0.800	0.800	0.654	0.767	0.533
Norway 63° N	1.000	1.000	0.972	0.971	1.000	1.000	1.000	0.964	1.000	0.929	0.964	0.969	0.833	0.735	1.000	0.750	0.750	0.588	0.500	0.912	0.767
Iceland	0.950	0.950	1.000	0.950	1.000	0.950	0.950	0.944	1.000	0.944	0.944	0.900	0.750	0.750	0.929	0.750	0.833	0.611	0.944	0.450	0.563
Norway 60° N	1.000	0.979	1.000	0.970	1.000	1.000	0.990	0.969	0.990	0.969	0.969	0.888	0.783	0.667	0.837	0.615	0.720	0.571	0.787	0.663	0.560
Norway 59° N	0.989	0.978	0.989	0.967	0.989	0.989	1.000	0.975	0.987	0.977	0.976	0.900	0.763	0.682	0.841	0.672	0.824	0.602	0.667	0.722	0.544
AtlanticXIIc	1.000	1.000	1.000	1.000	1.000	0.989	0.968	1.000	0.967	1.000	1.000	0.900	0.695	0.707	0.744	0.649	0.733	0.663	0.731	0.713	0.568
Celtic Sea	0.932	0.905	0.956	0.947	0.961	0.908	0.908	0.938	0.938	0.935	0.952	0.803	0.750	0.618	0.789	0.649	0.816	0.608	0.758	0.639	0.662
Flemish Cap	1.000	1.000	0.944	1.000	1.000	1.000	1.000	1.000	1.000	1.000	1.000	0.900	0.850	0.556	0.800	1.000	0.950	0.750	0.600	0.550	0.700
Atlantic 47° N	0.667	0.634	0.782	0.667	0.720	0.675	0.646	0.725	0.675	0.750	0.756	0.625	0.692	0.341	0.659	0.566	0.524	0.634	0.855	0.817	0.821
Bay of Biscay	0.593	0.571	0.654	0.411	0.571	0.589	0.611	0.435	0.563	0.455	0.458	0.537	0.674	0.407	0.554	0.660	0.346	0.625	0.891	0.768	0.839
Cantabrian Sea	0.233	0.597	0.536	0.371	0.500	0.250	0.419	0.313	0.525	0.281	0.342	0.484	0.700	0.210	0.452	0.532	0.355	0.552	0.933	0.806	0.774
Atlantic 40° N	0.500	0.533	0.745	0.500	0.622	0.500	0.571	0.489	0.556	0.500	0.478	0.459	0.648	0.327	0.604	0.574	0.385	0.656	0.857	0.875	0.833
Morocco	0.208	0.234	0.500	0.365	0.372	0.198	0.316	0.362	0.326	0.365	0.351	0.375	0.685	0.177	0.448	0.469	0.235	0.594	0.875	0.867	0.837
Alborán Sea	0.033	0.000	0.036	0.000	0.133	0.033	0.000	0.033	0.033	0.033	0.033	0.067	0.786	0.033	0.067	0.357	0.000	0.433	0.933	0.964	0.833
N Aegean Sea	0.000	0.047	0.009	0.000	0.183	0.000	0.000	0.000	0.000	0.010	0.000	0.538	0.526	0.000	0.087	0.208	0.000	0.236	1.000	1.000	0.873
N Euboean Gulf	0.178	0.860	1.000	0.273	0.315	0.151	0.424	0.057	0.182	0.273	0.024	0.548	0.659	0.163	0.625	0.685	0.185	0.305	0.957	0.967	0.973
Ionian Sea	0.054	0.105	0.309	0.000	0.213	0.064	0.000	0.011	0.000	0.043	0.000	0.500	0.408	0.044	0.222	0.479	0.000	0.117	0.979	1.000	0.950
Cretan Sea	0.000	0.081	0.011	0.000	0.170	0.000	0.000	0.000	0.012	0.012	0.000	0.602	0.553	0.000	0.034	0.181	0.000	0.198	1.000	0.989	0.763

*Note:* Greener colours indicate low values of allele frequency, increasing towards yellow, orange and finally red to indicate larger values of allele frequency.

### 4.5 Management Implications

In the context of ongoing climate change, reducing atmospheric CO_2_ concentrations is critical to mitigating global warming, a process in which the Biological Carbon Pump (BCP) plays a key role. The ecological importance of mesopelagic fishes stems from their contribution to carbon sequestration through the BCP (Hudson et al. 2014; St John et al. 2016) as well as from their role in supporting ecosystem services by serving as prey for a wide range of predators, including economically valuable species (Iglesias et al. 2023).

However, the large estimates of mesopelagic fish biomass—strongly debated (Pauly et al. 2021) around ranges of 1.8–16 Gt (Proud et al. 2018) and 2–19.5 Gt (Hidalgo and Browman 2019) —correspond to ~100 times the annual global fisheries catch, which stimulated the interest in the potential exploitation of this relatively intact putative resource, primarily as animal feed for aquaculture (Alvheim et al. 2020; Grimaldo et al. 2020) and to meet the increasing demand for fish oil (Tacon and Metian 2008). While commercial exploitation has so far yielded limited success (Prellezo 2019), trial fisheries targeting mesopelagic fish (including *M. muelleri* ) have recently been carried out by Norwegian vessels in the North Atlantic (Standal and Grimaldo 2021). Given the inherent risks of commercial exploitation, a precautionary approach is required to ensure that the ecological services provided by mesopelagic species are not compromised (Dişa et al. 2024). Accordingly, the ecological importance of *M. muelleri* must be rigorously evaluated before any harvesting is considered to prevent potentially irreversible consequences.

In the face of an eventual commercial exploitation, and to ensure sustainability, a compelling body of literature highlights the importance of accurately aligning genetic and biological units when defining fisheries stocks (e.g., Allendorf et al. 2008; Bernatchez et al. 2017; Cadrin et al. 2014; Funk et al. 2012; Hauser and Carvalho 2008; Kerr et al. 2017; Reiss et al. 2009; Waples et al. 2008). The identification of three distinct genetic populations of *M. muelleri* in the North Atlantic Ocean and Mediterranean basins provides essential background information. In addition, demographic properties should be mapped to provide a more comprehensive delineation of stocks ensuring that biologically meaningful units are aligned with management boundaries to inform a sustainable fishery.

Finally, genetic response to environmental changes can happen at a high pace in the marine realm. One century was enough to generate an extremely distinct population of European sprat after colonizing an artificially created brackish lake (Pettersson et al. 2024; Quintela et al. 2021). To project the response of *M. muelleri* into future climatic scenarios and aid in informing conservation strategies in the face of the ongoing climate change, it would be crucial to understand the influence of temperature in driving local adaptation using whole genome data.

## Funding

This work was supported by CISC–European Union sampling Programme; Norges Forskningsråd, HARMES project (project number 280546); MesoBED–Hellenic Foundation for Research and Innovation and the General Secretariat of Research and Innovation, 449; SUMMER EU H2020 Research and Innovation Programme, 817806; Norwegian Department for Trade and Fisheries; MEESO project – EU H2020 Research and Innovation Programme, 817669.

## Disclosure

Benefits generated: A research collaboration was developed with all the scientists from the countries providing genetic samples; all collaborators are included as co‐authors, the results of the research have been shared with the provider communities and the broader scientific community, and the research addresses a priority concern, in this case the conservation of organisms being studied. More broadly, our group is committed to international scientific partnerships, as well as institutional capacity building.

## Conflicts of Interest

The authors declare no conflicts of interest.

## Supporting information


**Table S1:** List of the 30 discarded SNPs together with the reasons for them to be removed.
**Table S2:** Compilation of pairwise *F*_ST_ matrices from total non‐filtered data till the 170 individually genotyped SNPs retained for analyses:
**Table S3:** Genetic differentiation between geographically explicit samples assessed with 170 SNPs: Heatmap of pairwise *F*_ST_ values in the bottom diagonal and corresponding *p*‐values after 10,000 permutations in the top diagonal, with the ones significantly different from zero after FDR correction highlighted in boldface type.
**Table S4:** Summary of information for loci involved in allele frequency clines.
**Table S5:** Summary of Mantel and Partial Mantel tests calculated conducting genetic distance (*F*_ST_).
**Figure S1:** *Maurolicus muelleri* : SNP mining using four samples in three different habitats.
**Figure S2:** Proportion of polymorphic loci per sample using different sets of SNPs during the process of filtering of pool genome data as well as for the individually genotyped individuals using the Sequenom.
**Figure S3:** Genotype accumulation curve calculated for the set of 170 polymorphic SNP loci using the total 863 individuals.
**Figure S4:** A posteriori analysis of STRUCTURE outcome for the set of 170 loci following Puechmaille and Evanno's statistics, respectively.
**Figure S5:** Barplot representing the proportion of individuals' ancestry to cluster at K3 to K5 after Bayesian clustering in STRUCTURE using the 170 total loci.
**Figure S6:** Examples of cline latitudinal patterns for STRUCTURE Q‐score (a) and locus P105 (b).
**Figure S7:** Detailed map of the studied area in the Greek Seas (a) and temperature at depth measured with CTD at the corresponding sampling sites (b).

## Data Availability

The genotype raw data used in this study is publicly accessible from the electronic archive of the Institute of Marine Research at https://hdl.handle.net/11250/3180126.

## References

[eva70188-bib-0001] Aarbakke, O. N. S. , A. Bucklin , C. Halsband , and F. Norrbin . 2014. “Comparative Phylogeography and Demographic History of Five Sibling Species of *Pseudocalanus* (Copepoda: Calanoida) in the North Atlantic Ocean.” Journal of Experimental Marine Biology and Ecology 461: 479–488. 10.1016/j.jembe.2014.10.006.

[eva70188-bib-0002] Allendorf, F. W. , P. R. England , G. Luikart , P. A. Ritchie , and N. Ryman . 2008. “Genetic Effects of Harvest on Wild Animal Populations.” Trends in Ecology & Evolution 23, no. 6: 327–337. 10.1016/j.tree.2008.02.008. 18439706

[eva70188-bib-0003] Alvarez, P. , N. Aldanondo , A. M. Wieczorek , et al. 2024. “Exploring Growth Patterns of *Maurolicus muelleri* Across Three Northeast Atlantic Regions.” Fishes 9, no. 7: 250. 10.3390/fishes9070250.

[eva70188-bib-0004] Alvheim, A. R. , M. Kjellevold , E. Strand , M. Sanden , and M. Wiech . 2020. “Mesopelagic Species and Their Potential Contribution to Food and Feed Security—A Case Study From Norway.” Food 9, no. 3: 344. 10.3390/foods9030344. PMC7142554 32188085

[eva70188-bib-0005] Baliño, B. 1993. “Winter Distribution and Migration of the Sound Scattering Layers, Zooplankton and Micronekton in Masfjorden, Western Norway.” Marine Ecology Progress Series 1–2: 35–50. 10.3354/meps045045.

[eva70188-bib-0006] Bañón, R. , J. C. Arronte , C. Rodríguez‐Cabello , C. G. Piñeiro , A. Punzón , and A. Serrano . 2016. “Commented Checklist of Marine Fishes From the Galicia Bank Seamount (NW Spain).” Zootaxa 4067, no. 3: 293–333. 10.11646/zootaxa.4067.3.2. 27395877

[eva70188-bib-0007] Bargelloni, L. , J. A. Alarcon , M. C. Alvarez , et al. 2003. “Discord in the Family Sparidae (Teleostei): Divergent Phylogeographical Patterns Across the Atlantic–Mediterranean Divide.” Journal of Evolutionary Biology 16, no. 6: 1149–1158. 10.1046/j.1420-9101.2003.00620.x. 14640406

[eva70188-bib-0008] Barros‐García, D. , E. Froufe , R. Bañón , J. C. Arronte , F. Baldó , and A. de Carlos . 2020. “Phylogeography Highlights Two Different Atlantic/Mediterranean Lineages and a Phenotypic Latitudinal Gradient for the Deep‐Sea Morid Codling *Lepidion lepidion* (Gadiformes: Moridae).” Deep Sea Research Part I: Oceanographic Research Papers 157: 103212. 10.1016/j.dsr.2019.103212.

[eva70188-bib-0009] Benestan, L. M. , Q. Rougemont , C. Senay , et al. 2021. “Population Genomics and History of Speciation Reveal Fishery Management Gaps in Two Related Redfish Species (*Sebastes mentella* and *Sebastes fasciatus* ).” Evolutionary Applications 14, no. 2: 588–606. 10.1111/eva.13143. 33664797 PMC7896722

[eva70188-bib-0010] Benjamini, Y. , and Y. Hochberg . 1995. “Controlling the False Discovery Rate: A Practical and Powerful Approach to Multiple Testing.” Journal of the Royal Statistical Society. Series B, Statistical Methodology 57, no. 1: 289–300. 10.1111/j.2517-6161.1995.tb02031.x.

[eva70188-bib-0011] Berg, P. R. , P. E. Jorde , K. A. Glover , et al. 2021. “Genetic Structuring in Atlantic Haddock Contrasts With Current Management Regimes.” ICES Journal of Marine Science 78, no. 1: 1–13. 10.1093/icesjms/fsaa204.

[eva70188-bib-0012] Bernal, A. , V. M. Tuset , and M. P. Olivar . 2023. “Multiple Approaches to the Trophic Role of Mesopelagic Fish Around the Iberian Peninsula.” Animals 13, no. 5: 886. 10.3390/ani13050886. 36899743 PMC10000212

[eva70188-bib-0013] Bernatchez, L. , M. Wellenreuther , C. Araneda , et al. 2017. “Harnessing the Power of Genomics to Secure the Future of Seafood.” Trends in Ecology & Evolution 32, no. 9: 665–680. 10.1016/j.tree.2017.06.010. 28818341

[eva70188-bib-0201] Besnier, F. , and K. A. Glover . 2013. “Parallel Structure: A R Package to Distribute Parallel Runs of the Population Genetics Program Structureon Multi‐core Computers.” PLoS ONE 8, no. 7: e70651. 10.1371/journal.pone.0070651. 23923012 PMC3726640

[eva70188-bib-0014] Bjelland, O. 1995. “Life‐History Tactics of Two Fjordic Populations of Maurolicus Muelleri.” (Ms. Master Thesis), University of Bergen, Bergen.

[eva70188-bib-0016] Boyd, P. W. 2015. “Toward Quantifying the Response of the Oceans' Biological Pump to Climate Change.” Frontiers in Marine Science 2: 77. 10.3389/fmars.2015.00077.

[eva70188-bib-0015] Boyd, P. W. , H. Claustre , M. Levy , D. A. Siegel , and T. Weber . 2019. “Multi‐Faceted Particle Pumps Drive Carbon Sequestration in the Ocean.” Nature 568, no. 7752: 327–335. 10.1038/s41586-019-1098-2. 30996317

[eva70188-bib-0017] Bremer, J. R. A. , J. Mejuto , T. W. Greig , and B. Ely . 1996. “Global Population Structure of the Swordfish ( *Xiphias gladius* L.) as Revealed by Analysis of the Mitochondrial DNA Control Region.” Journal of Experimental Marine Biology and Ecology 197, no. 2: 295–310. 10.1016/0022-0981(95)00164-6.

[eva70188-bib-0018] Cadrin, S. X. , L. A. Kerr , and S. Mariani . 2014. Stock Identification Methods: Applications in Fishery Science. Second ed. Academic Press.

[eva70188-bib-0019] Chen, S. , Y. Zhou , Y. Chen , and J. Gu . 2018. “Fastp: An Ultra‐Fast All‐In‐One FASTQ Preprocessor.” Bioinformatics 34, no. 17: i884–i890. 10.1093/bioinformatics/bty560. 30423086 PMC6129281

[eva70188-bib-0020] Cherel, Y. , C. Fontaine , P. Richard , and J.‐P. Labatc . 2010. “Isotopic Niches and Trophic Levels of Myctophid Fishes and Their Predators in the Southern Ocean.” Limnology and Oceanography 55, no. 1: 324–332. 10.4319/lo.2010.55.1.0324.

[eva70188-bib-0021] Dahle, G. , M. Quintela , T. Johansen , et al. 2018. “Analysis of Coastal Cod ( *Gadus morhua* L.) Sampled on Spawning Sites Reveals a Genetic Gradient Throughout Norway's Coastline.” BMC Genetics 19, no. 1: 42. 10.1186/s12863-018-0625-8. 29986643 PMC6036686

[eva70188-bib-0022] Dahms, C. , and S. S. Killen . 2023. “Temperature Change Effects on Marine Fish Range Shifts: A Meta‐Analysis of Ecological and Methodological Predictors.” Global Change Biology 29, no. 16: 4459–4479. 10.1111/gcb.16770. 37253462

[eva70188-bib-0023] de Van Putte, A. P. , J. K. J. Van Houdt , G. E. Maes , B. Hellemans , M. A. Collins , and F. A. M. Volckaert . 2012. “High Genetic Diversity and Connectivity in a Common Mesopelagic Fish of the Southern Ocean: The Myctophid *Electrona antarctica* .” Deep Sea Research Part II: Topical Studies in Oceanography 59: 199–207. 10.1016/j.dsr2.2011.05.011.

[eva70188-bib-0202] Derryberry, E. P. , G. E. Derryberry , J. M. Maley , and R. T. Brumfield . 2014. “HZAR: Hybrid Zone Analysis using an R Software Package.” Molecular Ecology Resources 14, no. 3: 652–663. 10.1111/1755-0998.12209. 24373504

[eva70188-bib-0024] Dişa, D. , E. Akoglu , and B. Salihoglu . 2024. “Exploitation of Mesopelagic Fish Stocks Can Impair the Biological Pump and Food Web Dynamics in the Ocean.” Frontiers in Marine Science 11: 1389941. 10.3389/fmars.2024.1389941.

[eva70188-bib-0025] Dorant, Y. , H. Cayuela , K. Wellband , et al. 2020. “Copy Number Variants Outperform SNPs to Reveal Genotype‐Temperature Association in a Marine Species.” Molecular Ecology 29, no. 24: 4765–4782. 10.1111/mec.15565. 32803780

[eva70188-bib-0026] Drakopoulos, P. G. , and A. Lascaratos . 1998. “A Preliminary Study on the Internal Tides of the Gulfs of Patras and Korinthos, Greece.” Continental Shelf Research 18, no. 12: 1517–1529. 10.1016/S0278-4343(98)00052-1.

[eva70188-bib-0027] Dray, S. , and A.‐B. Dufour . 2007. “The ade4 Package: Implementing the Duality Diagram for Ecologists.” Journal of Statistical Software 22, no. 4: 1–20. 10.18637/jss.v022.i04.

[eva70188-bib-0028] Drazen, J. C. , and T. T. Sutton . 2017. “Dining in the Deep: The Feeding Ecology of Deep‐Sea Fishes.” Annual Review of Marine Science 9: 337–366. 10.1146/annurev-marine-010816-060543. 27814034

[eva70188-bib-0029] Ducklow, H. 2001. “Upper Ocean Carbon Export and the Biological Pump.” Oceanography 14: 50–54. 10.5670/oceanog.2001.06.

[eva70188-bib-0030] Duranton, M. , F. Allal , C. Fraïsse , N. Bierne , F. Bonhomme , and P. A. Gagnaire . 2018. “The Origin and Remolding of Genomic Islands of Differentiation in the European Sea Bass.” Nature Communications 9, no. 1: 2518. 10.1038/s41467-018-04963-6. PMC6023918 29955054

[eva70188-bib-0301] Evanno, G. , S. Regnaut , and J. Goudet . 2005. “Detecting the Number of Clusters of Individuals Using the Software STRUCTURE: A Simulation Study.” Molecular Ecology 14, no. 8: 2611–2620. 10.1111/j.1365-294X.2005.02553.x. 15969739

[eva70188-bib-0031] Excoffier, L. , G. Laval , and S. Schneider . 2005. “Arlequin ver. 3.0: An Integrated Software Package for Population Genetics Data Analysis.” Evolutionary Bioinformatics Online 1: 47–50. 10.1177/117693430500100003. PMC2658868 19325852

[eva70188-bib-0032] Falk, A. H. , and O.‐A. Nøst . 2013. “Oppstrømming av Dyphavsvann – Litteraturstudie av Oppstrømming Utenfor Salten/Lofoten/Vesterålen. Akvaplan‐niva Rapport nr 6311‐01. 32.”

[eva70188-bib-0033] FAO . 2020. Report of the Working Group on the Assessment of Small Pelagic Fish of Northwest Africa Casablanca, Morocco, 8–13 July 2019. Rapport de Groupe de Travail Sur L'évaluation Des Petits Pêlagiques Au Large de L'afrique Nord‐Occidentale Casablanca, Maroc, 8–13 Juillet 2019. Fishery Committee for the Eastern Central Atlantic (CECAF)/Comité des Pêches pour l'Atlantique Centre‐Est (COPACE). RFAO Fisheries and Aquaculture Report No. 1309/FAO, Rapport sur les Pêches et l'aquaculture no 1309. FAO. 10.4060/ca9562b.

[eva70188-bib-0034] Farmer, D. M. , and H. J. Freeland . 1983. “The Physical Oceanography of Fjords.” Progress in Oceanography 12, no. 2: 147–219. 10.1016/0079-6611(83)90004-6.

[eva70188-bib-0035] Fontaine, M. C. 2016. “Chapter Eleven – Harbour Porpoises, *Phocoena phocoena*, in the Mediterranean Sea and Adjacent Regions: Biogeographic Relicts of the Last Glacial Period.” In Advances in Marine Biology, edited by G. Notarbartolo Di Sciara , M. Podestà , and B. E. Curry , vol. 75, 333–358. Academic Press. 10.1016/bs.amb.2016.08.006 27770989

[eva70188-bib-0036] Froese, R. , N. Steiner , E. Papaioannou , L. MacNeil , T. B. H. Reusch , and M. Scotti . 2025. “Systemic Failure of European Fisheries Management.” Science 388, no. 6749: 826–828. 40403071 10.1126/science.adv4341

[eva70188-bib-0037] Funk, W. C. , J. K. McKay , P. A. Hohenlohe , and F. W. Allendorf . 2012. “Harnessing Genomics for Delineating Conservation Units.” Trends in Ecology & Evolution 27, no. 9: 489–496. 10.1016/j.tree.2012.05.012. 22727017 PMC4185076

[eva70188-bib-0038] Gabriel, S. , L. Ziaugra , and D. Tabbaa . 2009. “SNP Genotyping Using the Sequenom MassARRAY iPLEX Platform.” Current Protocols in Human Genetics 60, no. 1: 12. 10.1002/0471142905.hg0212s60. 19170031

[eva70188-bib-0039] Galarza, J. A. , J. Carreras‐Carbonell , E. Macpherson , et al. 2009. “The Influence of Oceanographic Fronts and Early‐Life‐History Traits on Connectivity Among Littoral Fish Species.” Proceedings of the National Academy of Sciences 106, no. 5: 1473–1478. 10.1073/pnas.0806804106. PMC2629446 19164518

[eva70188-bib-0040] Galarza, J. A. , G. F. Turner , E. Macpherson , and C. Rico . 2009. “Patterns of Genetic Differentiation Between Two Co‐Occurring Demersal Species: The Red Mullet ( *Mullus barbatus* ) and the Striped Red Mullet ( *Mullus surmuletus* ).” Canadian Journal of Fisheries and Aquatic Sciences 66, no. 9: 1478–1490. 10.1139/f09-098.

[eva70188-bib-0041] Giske, J. , D. L. Aksnes , B. M. Baliño , et al. 1990. “Vertical Distribution and Trophic Interactions of Zooplankton and Fish in Masfjorden, Norway.” Sarsia 75: 65–81. 10.1080/00364827.1990.10413442.

[eva70188-bib-0042] Gjøsæter, J. , and K. Kawaguchi . 1980. A Review of the World Resources of Mesopelagic Fish. FAO Fish. Tech. Pap. No. 193, 151. Food And Agriculture Organization Of The United Nations.

[eva70188-bib-0043] Goodson, M. S. , J. Giske , and R. Rosland . 1995. “Growth and Ovarian Development of *Maurolicus muelleri* During Spring.” Marine Biology 124, no. 2: 185–195. 10.1007/BF00347122.

[eva70188-bib-0044] Grimaldo, E. , L. Grimsmo , P. Álvarez , et al. 2020. “Investigating the Potential for a Commercial Fishery in the Northeast Atlantic Utilizing Mesopelagic Species.” ICES Journal of Marine Science 77, no. 7–8: 2541–2556. 10.1093/icesjms/fsaa114.

[eva70188-bib-0045] Hansen, A. , J.‐I. Westgaard , G. Søvik , et al. 2021. “Genetic Differentiation Between Inshore and Offshore Populations of Northern Shrimp ( *Pandalus borealis* ).” ICES Journal of Marine Science 78, no. 9: 3135–3146. 10.1093/icesjms/fsab181.

[eva70188-bib-0046] Harmelin, J.‐G. , and J.‐L. d'Hondt . 1993. “Transfers of Bryozoan Species Between the Atlantic Ocean and the Mediterranean Sea via the Strait of Gibraltar.” Oceanologica Acta 16, no. 1: 63–72.

[eva70188-bib-0047] Hauser, L. , and G. R. Carvalho . 2008. “Paradigm Shifts in Marine Fisheries Genetics: Ugly Hypotheses Slain by Beautiful Facts.” Fish and Fisheries 9, no. 4: 333–362. 10.1111/j.1467-2979.2008.00299.x.

[eva70188-bib-0048] Hays, G. C. 2003. “A Review of the Adaptive Significance and Ecosystem Consequences of Zooplankton Diel Vertical Migrations.” Hydrobiologia 503, no. 1: 163–170. 10.1023/B:HYDR.0000008476.23617.b0.

[eva70188-bib-0049] Hidaka, K. , K. Kawaguchi , M. Murakami , and M. Takahashi . 2001. “Downward Transport of Organic Carbon by Diel Migratory Micronekton in the Western Equatorial Pacific: Its Quantitative and Qualitative Importance.” Deep Sea Research, Part I: Oceanographic Research Papers 48, no. 8: 1923–1939. 10.1016/S0967-0637(01)00003-6.

[eva70188-bib-0050] Hidalgo, M. , and H. I. Browman . 2019. “Developing the Knowledge Base Needed to Sustainably Manage Mesopelagic Resources.” ICES Journal of Marine Science 76, no. 3: 609–615. 10.1093/icesjms/fsz067.

[eva70188-bib-0051] Hudson, J. M. , D. K. Steinberg , T. T. Sutton , J. E. Graves , and R. J. Latour . 2014. “Myctophid Feeding Ecology and Carbon Transport Along the Northern Mid‐Atlantic Ridge.” Deep Sea Research Part I: Oceanographic Research Papers 93: 104–116. 10.1016/j.dsr.2014.07.002.

[eva70188-bib-0052] Iglesias, I. S. , J. A. Santora , J. Fiechter , and J. C. Field . 2023. “Mesopelagic Fishes Are Important Prey for a Diversity of Predators.” Frontiers in Marine Science 10: 88. 10.3389/fmars.2023.1220088.

[eva70188-bib-0053] Irigoien, X. , T. A. Klevjer , A. Røstad , et al. 2014. “Large Mesopelagic Fishes Biomass and Trophic Efficiency in the Open Ocean.” Nature Communications 5, no. 1: 3271. 10.1038/ncomms4271. PMC3926006 24509953

[eva70188-bib-0054] Jakobsson, M. , and N. A. Rosenberg . 2007. “CLUMPP: A Cluster Matching and Permutation Program for Dealing With Label Switching and Multimodality in Analysis of Population Structure.” Bioinformatics 23, no. 14: 1801–1806. 10.1093/bioinformatics/btm233. 17485429

[eva70188-bib-0055] Jenkins, T. L. , C. D. Ellis , A. Triantafyllidis , and J. R. Stevens . 2019. “Single Nucleotide Polymorphisms Reveal a Genetic Cline Across the North‐East Atlantic and Enable Powerful Population Assignment in the European Lobster.” Evolutionary Applications 12, no. 10: 1881–1899. 10.1111/eva.12849. 31700533 PMC6824076

[eva70188-bib-0056] Johannesson, K. , and C. Andre . 2006. “Life on the Margin: Genetic Isolation and Diversity Loss in a Peripheral Marine Ecosystem, the Baltic Sea.” Molecular Ecology 15, no. 8: 2013–2029. 10.1111/j.1365-294X.2006.02919.x. 16780421

[eva70188-bib-0057] Johannesson, K. , A.‐K. Ring , K. B. Johannesson , E. Renborg , P. R. Jonsson , and J. N. Havenhand . 2018. “Oceanographic Barriers to Gene Flow Promote Genetic Subdivision of the Tunicate *Ciona intestinalis* in a North Sea Archipelago.” Marine Biology 165, no. 8: 126. 10.1007/s00227-018-3388-x. 30100627 PMC6061499

[eva70188-bib-0058] Jombart, T. 2008. “ *Adegenet*: A R Package for the Multivariate Analysis of Genetic Markers.” Bioinformatics 24: 1403–1405. 10.1093/bioinformatics/btn129. 18397895

[eva70188-bib-0059] Jombart, T. , and C. Collins . 2015. “A Tutorial for Discriminant Analysis of Principal Components (DAPC) Using Adegenet 2.0.0.” https://adegenet.r‐forge.r‐project.org/files/tutorial‐dapc.pdf.

[eva70188-bib-0060] Jombart, T. , S. Devillard , and F. Balloux . 2010. “Discriminant Analysis of Principal Components: A New Method for the Analysis of Genetically Structured Populations.” BMC Genetics 11, no. 1: 94. 10.1186/1471-2156-11-94. 20950446 PMC2973851

[eva70188-bib-0061] Jorde, P. E. , M. B. O. Huserbråten , B. B. Seliussen , et al. 2021. “The Making of a Genetic Cline: Introgression of Oceanic Genes Into Coastal Cod Populations in the Northeast Atlantic.” Canadian Journal of Fisheries and Aquatic Sciences 78, no. 7: 958–968. 10.1139/cjfas-2020-0380.

[eva70188-bib-0062] Kaartvedt, S. , A. Røstad , T. Klevjer , and A. Staby . 2009. “Use of Bottom‐Mounted Echo Sounders in Exploring Behavior of Mesopelagic Fishes.” Marine Ecology Progress Series 395: 109–118. 10.3354/meps08174.

[eva70188-bib-0063] Kamvar, Z. N. , J. F. Tabima , and N. J. Grünwald . 2014. “Poppr: An R Package for Genetic Analysis of Populations With Clonal, Partially Clonal, and/or Sexual Reproduction.” PeerJ 2: e281. 10.7717/peerj.281. 24688859 PMC3961149

[eva70188-bib-0064] Kapelonis, Z. , A. Siapatis , A. Machias , et al. 2023. “Seasonal Patterns in the Mesopelagic Fish Community and Associated Deep Scattering Layers of an Enclosed Deep Basin.” Scientific Reports 13, no. 1: 17890. 10.1038/s41598-023-44765-5. 37857721 PMC10587179

[eva70188-bib-0065] Kerr, L. A. , N. T. Hintzen , S. X. Cadrin , et al. 2017. “Lessons Learned From Practical Approaches to Reconcile Mismatches Between Biological Population Structure and Stock Units of Marine Fish.” ICES Journal of Marine Science 74, no. 6: 1708–1722. 10.1093/icesjms/fsw188.

[eva70188-bib-0067] Knaus, B. J. , and N. J. Grünwald . 2016. “VcfR: A Package to Manipulate and Visualize VCF Format Data in R.” *bioRxiv*. 10.1101/041277. 27401132

[eva70188-bib-0066] Knaus, B. J. , and N. J. Grünwald . 2017. “Vcfr: A Package to Manipulate and Visualize Variant Call Format Data in R.” Molecular Ecology Resources 17, no. 1: 44–53. 10.1111/1755-0998.12549. 27401132

[eva70188-bib-0068] Knutsen, H. , P. E. Jorde , J. A. Hutchings , et al. 2018. “Stable Coexistence of Genetically Divergent Atlantic Cod Ecotypes at Multiple Spatial Scales.” Evolutionary Applications 11, no. 9: 1527–1539. 10.1111/eva.12640. 30344625 PMC6183466

[eva70188-bib-0069] Knutsen, H. , P. E. Jorde , H. Sannæs , et al. 2009. “Bathymetric Barriers Promoting Genetic Structure in the Deepwater Demersal Fish Tusk (*Brosme brosme*).” Molecular Ecology 18, no. 15: 3151–3162. 10.1111/j.1365-294X.2009.04253.x. 19549108

[eva70188-bib-0070] Kojima, S. , M. Moku , and K. Kawaguchi . 2009. “Genetic Diversity and Population Structure of Three Dominant Myctophid Fishes (* Diaphus theta, Stenobrachius leucopsarus*, and *S. nannochir* ) in the North Pacific Ocean.” Journal of Oceanography 65, no. 2: 187–193. 10.1007/s10872-009-0018-8.

[eva70188-bib-0072] Kristoffersen, J. B. , and A. G. V. Salvanes . 1998. “Life History of *Maurolicus muelleri* in Fjordic and Oceanic Environments.” Journal of Fish Biology 53, no. 6: 1324–1341. 10.1111/j.1095-8649.1998.tb00252.x.

[eva70188-bib-0073] Kristoffersen, J. B. , and A. G. V. Salvanes . 2001. “Sexual Size Dimorphism and Sex Ratio in Müller's Pearlside (*Maurolicus muelleri*).” Marine Biology 138, no. 6: 1087–1092. 10.1007/s002270000529.

[eva70188-bib-0071] Kristoffersen, J. B. , and A. G. V. Salvanes . 2009. “Distribution, Growth, and Population Genetics of the Glacier Lanternfish (*Benthosema glaciale*) in Norwegian Waters: Contrasting Patterns in Fjords and the Ocean.” Marine Biology Research 5, no. 6: 596–604. 10.1080/17451000903042479.

[eva70188-bib-0074] Lapègue, S. , C. Reisser , E. Harrang , S. Heurtebise , and N. Bierne . 2023. “Genetic Parallelism Between European Flat Oyster Populations at the Edge of Their Natural Range.” Evolutionary Applications 16, no. 2: 393–407. 10.1111/eva.13449. 36793680 PMC9923475

[eva70188-bib-0075] Le Moan, A. , P.‐A. Gagnaire , and F. Bonhomme . 2016. “Parallel Genetic Divergence Among Coastal–Marine Ecotype Pairs of European Anchovy Explained by Differential Introgression After Secondary Contact.” Molecular Ecology 25, no. 13: 3187–3202. 10.1111/mec.13627. 27027737

[eva70188-bib-0076] Li, D. , C.‐M. Liu , R. Luo , K. Sadakane , and T.‐W. Lam . 2015. “MEGAHIT: An Ultra‐Fast Single‐Node Solution for Large and Complex Metagenomics Assembly via Succinct *de Bruijn* Graph.” Bioinformatics 31, no. 10: 1674–1676. 10.1093/bioinformatics/btv033. 25609793

[eva70188-bib-0077] Li, D. , R. Luo , C. M. Liu , et al. 2016. “MEGAHIT v1.0: A Fast and Scalable Metagenome Assembler Driven by Advanced Methodologies and Community Practices.” Methods 102: 3–11. 10.1016/j.ymeth.2016.02.020. 27012178

[eva70188-bib-0078] Li, H. , and R. Durbin . 2010. “Fast and Accurate Long‐Read Alignment With Burrows–Wheeler Transform.” Bioinformatics 26, no. 5: 589–595. 10.1093/bioinformatics/btp698. 20080505 PMC2828108

[eva70188-bib-0079] Li, H. , B. Handsaker , A. Wysoker , et al. 2009. “The Sequence Alignment/Map Format and SAMtools.” Bioinformatics 25, no. 16: 2078–2079. 10.1093/bioinformatics/btp352. 19505943 PMC2723002

[eva70188-bib-0080] Li, H. 2011. “A Statistical Framework for SNP Calling, Mutation Discovery, Association Mapping and Population Genetical Parameter Estimation From Sequencing Data.” Bioinformatics 27, no. 21: 2987–2993. 10.1093/bioinformatics/btr509. 21903627 PMC3198575

[eva70188-bib-0081] Li, Y.‐L. , and J.‐X. Liu . 2018. “StructureSelector: A Web‐Based Software to Select and Visualize the Optimal Number of Clusters Using Multiple Methods.” Molecular Ecology Resources 18, no. 1: 176–177. 10.1111/1755-0998.12719. 28921901

[eva70188-bib-0082] Lischer, H. E. L. , and L. Excoffier . 2012. “PGDSpider: An Automated Data Conversion Tool for Connecting Population Genetics and Genomics Programs.” Bioinformatics 28, no. 2: 298–299. 10.1093/bioinformatics/btr642. 22110245

[eva70188-bib-0083] Mantel, N. 1967. “The Detection of Disease of Clustering and a Generalized Regression Approach.” Cancer Research 27, no. 2: 209–220. 6018555

[eva70188-bib-0084] Marie, A. D. , C. Lejeusne , E. Karapatsiou , et al. 2016. “Implications for Management and Conservation of the Population Genetic Structure of the Wedge Clam *Donax trunculus* Across Two Biogeographic Boundaries.” Scientific Reports 6, no. 1: 39152. 10.1038/srep39152. 27991535 PMC5171699

[eva70188-bib-0085] Matschiner, M. , R. Hanel , and W. Salzburger . 2009. “Gene Flow by Larval Dispersal in the Antarctic Notothenioid Fish *Gobionotothen gibberifrons* .” Molecular Ecology 18, no. 12: 2574–2587. 10.1111/j.1365-294X.2009.04220.x. 19457182

[eva70188-bib-0086] Meyer, L. , P. Barry , F. Riquet , et al. 2024. “Divergence and Gene Flow History at Two Large Chromosomal Inversions Underlying Ecotype Differentiation in the Long‐Snouted Seahorse.” Molecular Ecology 33: e17277. 10.1111/mec.17277. 38279695

[eva70188-bib-0087] Miller, J. M. , C. I. Cullingham , and R. M. Peery . 2020. “The Influence of a Priori Grouping on Inference of Genetic Clusters: Simulation Study and Literature Review of the DAPC Method.” Heredity 125, no. 5: 269–280. 10.1038/s41437-020-0348-2. 32753664 PMC7553915

[eva70188-bib-0088] Nesbø, C. L. , E. K. Rueness , S. A. Iversen , D. W. Skagen , and K. S. Jakobsen . 2000. “Phylogeography and Population History of Atlantic Mackerel ( *Scomber scombrus* L.): A Genealogical Approach Reveals Genetic Structuring Among the Eastern Atlantic Stocks.” Proceedings of the Royal Society of London B 267, no. 1440: 281–292. 10.1098/rspb.2000.0998. PMC1690521 10714883

[eva70188-bib-0089] Nowicki, M. , T. DeVries , and D. A. Siegel . 2022. “Quantifying the Carbon Export and Sequestration Pathways of the Ocean's Biological Carbon Pump.” Global Biogeochemical Cycles 36, no. 3: e2021GB007083. 10.1029/2021GB007083.

[eva70188-bib-0090] Olivar, M. P. , A. Castellón , A. Sabatés , et al. 2022. “Variation in Mesopelagic Fish Community Composition and Structure Between Mediterranean and Atlantic Waters Around the Iberian Peninsula.” Frontiers in Marine Science 9: 717. 10.3389/fmars.2022.1028717.

[eva70188-bib-0091] Olivar, M. P. , T. Contreras , P. A. Hulley , et al. 2018. “Variation in the Diel Vertical Distributions of Larvae and Transforming Stages of Oceanic Fishes Across the Tropical and Equatorial Atlantic.” Progress in Oceanography 160: 83–100. 10.1016/j.pocean.2017.12.005.

[eva70188-bib-0092] Palacios‐Abrantes, J. , T. L. Frölicher , G. Reygondeau , et al. 2022. “Timing and Magnitude of Climate‐Driven Range Shifts in Transboundary Fish Stocks Challenge Their Management.” Global Change Biology 28, no. 7: 2312–2326. 10.1111/gcb.16058. 35040239 PMC9302671

[eva70188-bib-0093] Pampoulie, C. , A. K. Daníelsdóttir , M. Storr‐Paulsen , H. Hovgård , E. Hjörleifsson , and B. Æ. Steinarsson . 2011. “Neutral and Nonneutral Genetic Markers Revealed the Presence of Inshore and Offshore Stock Components of Atlantic Cod in Greenland Waters.” Transactions of the American Fisheries Society 140, no. 2: 307–319. 10.1080/00028487.2011.567850.

[eva70188-bib-0094] Pante, E. , and B. Simon‐Bouhet . 2013. “Marmap: A Package for Importing, Plotting and Analyzing Bathymetric and Topographic Data in R.” PLoS One 8, no. 9: e73051. 10.1371/journal.pone.0073051. 24019892 PMC3760912

[eva70188-bib-0095] Patarnello, T. , F. A. M. J. Volckaert , and R. Castilho . 2007. “Pillars of Hercules: Is the Atlantic–Mediterranean Transition a Phylogeographical Break?” Molecular Ecology 16, no. 21: 4426–4444. 10.1111/j.1365-294X.2007.03477.x. 17908222

[eva70188-bib-0096] Pauly, D. , C. Piroddi , L. Hood , et al. 2021. “The Biology of Mesopelagic Fishes and Their Catches (1950–2018) by Commercial and Experimental Fisheries.” Journal of Marine Science and Engineering 9, no. 10: 1057. 10.3390/jmse9101057.

[eva70188-bib-0097] Peakall, R. , and P. E. Smouse . 2006. “GenAlEx 6: Genetic Analysis in Excel. Population Genetic Software for Teaching and Research.” Molecular Ecology Notes 6, no. 1: 288–295. 10.1111/j.1471-8286.2005.01155.x. PMC3463245 22820204

[eva70188-bib-0098] Pereira, P. , J. Teixeira , and G. Velo‐Antón . 2018. “Allele Surfing Shaped the Genetic Structure of the European Pond Turtle via Colonization and Population Expansion Across the Iberian Peninsula From Africa.” Journal of Biogeography 45, no. 9: 2202–2215. 10.1111/jbi.13412.

[eva70188-bib-0099] Pérez‐Losada, M. , A. Guerra , G. R. Carvalho , A. Sanjuan , and P. W. Shaw . 2002. “Extensive Population Subdivision of the Cuttlefish *Sepia officinalis* (Mollusca: Cephalopoda) Around the Iberian Peninsula Indicated by Microsatellite DNA Variation.” Heredity 89, no. 6: 417–424. 10.1038/sj.hdy.6800160. 12466983

[eva70188-bib-0100] Pettersson, M. E. , M. Quintela , F. Besnier , et al. 2024. “Limited Parallelism in Genetic Adaptation to Brackish Water Bodies in European Sprat and Atlantic Herring.” Genome Biology and Evolution 16, no. 7: evae133. 10.1093/gbe/evae133. 38918882 PMC11226789

[eva70188-bib-0203] Pinsky, M. L. , and S. R. Palumbi . 2014. “Meta‐analysis Reveals Lower Genetic Diversity in Overfished Populations.” Molecular Ecology 23, no. 1: 29–39. 10.1111/mec.12509. 24372754

[eva70188-bib-0101] Prellezo, R. 2019. “Exploring the Economic Viability of a Mesopelagic Fishery in the Bay of Biscay.” ICES Journal of Marine Science 76, no. 3: 771–779. 10.1093/icesjms/fsy001.

[eva70188-bib-0102] Pritchard, J. K. , M. Stephens , and P. Donnelly . 2000. “Inference of Population Structure Using Multilocus Genotype Data.” Genetics 155, no. 2: 945–959. 10.1093/genetics/155.2.945. 10835412 PMC1461096

[eva70188-bib-0103] Proud, R. , N. O. Handegard , R. J. Kloser , M. J. Cox , and A. S. Brierley . 2018. “From Siphonophores to Deep Scattering Layers: Uncertainty Ranges for the Estimation of Global Mesopelagic Fish Biomass.” ICES Journal of Marine Science 76, no. 3: 718–733. 10.1093/icesjms/fsy037.

[eva70188-bib-0104] Puechmaille, S. J. 2016. “The Program Structure Does Not Reliably Recover the Correct Population Structure When Sampling Is Uneven: Subsampling and New Estimators Alleviate the Problem.” Molecular Ecology Resources 16, no. 3: 608–627. 10.1111/1755-0998.12512. 26856252

[eva70188-bib-0105] Quintela, M. , E. García‐Seoane , G. Dahle , et al. 2024. “Genetics in the Ocean's Twilight Zone: Population Structure of the Glacier Lanternfish Across Its Distribution Range.” Evolutionary Applications 17: e70032. 10.1111/eva.70032. 39513049 PMC11540841

[eva70188-bib-0106] Quintela, M. , C. Kvamme , D. Bekkevold , et al. 2020. “Genetic Analysis Redraws the Management Boundaries for the European Sprat.” Evolutionary Applications 13: 1906–1922. 10.1111/eva.12942. 32908594 PMC7463317

[eva70188-bib-0107] Quintela, M. , À. Richter‐Boix , D. Bekkevold , et al. 2021. “Genetic Response to Human‐Induced Habitat Changes in the Marine Environment: A Century of Evolution of European Sprat in Landvikvannet, Norway.” Ecology and Evolution 11, no. 4: 1691–1718. 10.1002/ece3.7160. 33613998 PMC7882954

[eva70188-bib-0108] Rábade Uberos, S. , A. R. Vergara Castaño , R. Domínguez‐Petit , and F. Saborido‐Rey . 2021. “Larval Fish Community in the Northwestern Iberian Upwelling System During the Summer Period.” Oceans 2, no. 4: 700–722. 10.3390/oceans2040040.

[eva70188-bib-0109] Ramfos, A. , S. Somarakis , C. Koutsikopoulos , and N. Fragopoulu . 2005. “Summer Mesozooplankton Distribution in Coastal Waters of Central Greece (Eastern Mediterranean). I. Hydrology and Group Composition.” Journal of the Marine Biological Association of the United Kingdom 85, no. 4: 755–764. 10.1017/S0025315405011665.

[eva70188-bib-0110] Rasmussen, O. I. , and J. Giske . 1994. “Life‐History Parameters and Vertical Distribution of *Maurolicus muelleri* in Masfjorden in Summer.” Marine Biology 120, no. 4: 649–664. 10.1007/BF00350086.

[eva70188-bib-0111] Rees, D. J. , I. Byrkjedal , and T. T. Sutton . 2017. “Pruning the Pearlsides: Reconciling Morphology and Molecules in Mesopelagic Fishes (*Maurolicus*: Sternoptychidae).” Deep Sea Research Part II: Topical Studies in Oceanography 137: 246–257. 10.1016/j.dsr2.2016.04.024.

[eva70188-bib-0112] Rees, D. J. , J. Y. Poulsen , T. T. Sutton , P. A. S. Costa , and M. F. Landaeta . 2020. “Global Phylogeography Suggests Extensive Eucosmopolitanism in Mesopelagic Fishes (*Maurolicus*: Sternoptychidae).” Scientific Reports 10, no. 1: 20544. 10.1038/s41598-020-77528-7. 33239750 PMC7689477

[eva70188-bib-0113] Reiss, H. , G. Hoarau , M. Dickey‐Collas , and W. J. Wolff . 2009. “Genetic Population Structure of Marine Fish: Mismatch Between Biological and Fisheries Management Units.” Fish and Fisheries 10, no. 4: 361–395. 10.1111/j.1467-2979.2008.00324.x.

[eva70188-bib-0114] Reuschel, S. , J. A. Cuesta , and C. D. Schubart . 2010. “Marine Biogeographic Boundaries and Human Introduction Along the European Coast Revealed by Phylogeography of the Prawn *Palaemon elegans* .” Molecular Phylogenetics and Evolution 55, no. 3: 765–775. 10.1016/j.ympev.2010.03.021. 20307676

[eva70188-bib-0115] Riesgo, A. , S. Taboada , R. Pérez‐Portela , et al. 2019. “Genetic Diversity, Connectivity and Gene Flow Along the Distribution of the Emblematic Atlanto‐Mediterranean Sponge *Petrosia ficiformis* (Haplosclerida, Demospongiae).” BMC Evolutionary Biology 19, no. 1: 24. 10.1186/s12862-018-1343-6. 30651060 PMC6335727

[eva70188-bib-0116] Riquet, F. , C. Liautard‐Haag , L. Woodall , et al. 2019. “Parallel Pattern of Differentiation at a Genomic Island Shared Between Clinal and Mosaic Hybrid Zones in a Complex of Cryptic Seahorse Lineages.” Evolution 73, no. 4: 817–835. 10.1111/evo.13696. 30854632

[eva70188-bib-0117] Robinet, T. , V. Roussel , K. Cheze , and P. A. Gagnaire . 2020. “Spatial Gradients of Introgressed Ancestry Reveal Cryptic Connectivity Patterns in a High Gene Flow Marine Fish.” Molecular Ecology 29, no. 20: 3857–3871. 10.1111/mec.15611. 32853456

[eva70188-bib-0118] Robinson, C. , D. K. Steinberg , T. R. Anderson , et al. 2010. “Mesopelagic Zone Ecology and Biogeochemistry – A Synthesis.” Deep Sea Research Part II: Topical Studies in Oceanography 57, no. 16: 1504–1518. 10.1016/j.dsr2.2010.02.018.

[eva70188-bib-0119] Rodríguez‐Ezpeleta, N. , P. Álvarez , and X. Irigoien . 2017. “Genetic Diversity and Connectivity in *Maurolicus muelleri* in the Bay of Biscay Inferred From Thousands of SNP Markers.” Frontiers in Genetics 8: 195. 10.3389/fgene.2017.00195. 29234350 PMC5712365

[eva70188-bib-0120] Rosenberg, M. S. , and C. D. Anderson . 2011. “PASSaGE: Pattern Analysis, Spatial Statistics and Geographic Exegesis. Version 2.” Methods in Ecology and Evolution 2, no. 3: 229–232. 10.1111/j.2041-210X.2010.00081.x.

[eva70188-bib-0121] Rousset, F. 2008. “GENEPOP'007: A Complete Re‐Implementation of the Genepop Software for Windows and Linux.” Molecular Ecology Resources 8, no. 1: 103–106. 10.1111/j.1471-8286.2007.01931.x. 21585727

[eva70188-bib-0122] Rousset, F. 1997. “Genetic Differentiation and Estimation of Gene Flow From F‐Statistics Under Isolation by Distance.” Genetics 145, no. 4: 1219–1228. 10.1093/genetics/145.4.1219. 9093870 PMC1207888

[eva70188-bib-0123] Ruzzante, D. E. , C. T. Taggart , D. Cook , and S. V. Goddard . 1997. “Genetic Differentiation Between Inshore and Offshore Atlantic Cod ( *Gadus morhua* ) off Newfoundland: A Test and Evidence of Temporal Stability.” Canadian Journal of Fisheries and Aquatic Sciences 54, no. 11: 2700–2708. 10.1139/f97-170.

[eva70188-bib-0124] Salvanes, A. G. V. , and B. M. Stockley . 1996. “Spatial Variation of Growth and Gonadal Developments of *Maurolicus muelleri* in the Norwegian Sea and in a Norwegian Fjord.” Marine Biology 126, no. 2: 321–332. 10.1007/BF00347456.

[eva70188-bib-0125] Sánchez‐Garrido, J. C. , and I. Nadal . 2022. “The Alboran Sea Circulation and Its Biological Response: A Review.” Frontiers in Marine Science 9: 15. 10.3389/fmars.2022.933390.

[eva70188-bib-0126] Sanders, R. , S. A. Henson , M. Koski , et al. 2014. “The Biological Carbon Pump in the North Atlantic.” Progress in Oceanography 129: 200–218. 10.1016/j.pocean.2014.05.005.

[eva70188-bib-0127] Sarropoulou, X. , D. Tsaparis , K. Tsagarakis , N. Badouvas , and C. S. Tsigenopoulos . 2022. “Different Patterns of Population Structure and Genetic Diversity of Three Mesopelagic Fishes in the Greek Seas.” Mediterranean Marine Science 23, no. 3: 536–545. 10.12681/mms.28567.

[eva70188-bib-0128] Schunter, C. , J. Carreras‐Carbonell , E. Macpherson , et al. 2011. “Matching Genetics With Oceanography: Directional Gene Flow in a Mediterranean Fish Species.” Molecular Ecology 20, no. 24: 5167–5181. 10.1111/j.1365-294X.2011.05355.x. 22097887

[eva70188-bib-0129] Shreeve, R. S. , M. A. Collins , G. A. Tarling , C. E. Main , P. Ward , and N. M. Johnston . 2009. “Feeding Ecology of Myctophid Fishes in the Northern Scotia Sea.” Marine Ecology Progress Series 386: 221–236. 10.3354/meps08064.

[eva70188-bib-0130] Sigman, D. M. , and G. H. Haug . 2006. “The Biological Pump in the Past.” In Treatise on Geochemistry. The Oceans and Marine Geochemistry, edited by H. Elderfield , vol. 6, 491–528. Elsevier.

[eva70188-bib-0131] Slatkin, M. 1993. “Isolation by Distance in Equilibrium and Non‐Equilibrium Populations.” Evolution 47, no. 1: 264–279. 10.1111/j.1558-5646.1993.tb01215.x. 28568097

[eva70188-bib-0132] Somarakis, S. , S. Isari , and A. Machias . 2011. “Larval Fish Assemblages in Coastal Waters of Central Greece: Reflections of Topographic and Oceanographic Heterogeneity.” Scientia Marina 75, no. 3: 605–618. 10.3989/scimar.2011.75n3605.

[eva70188-bib-0133] St John, M. A. , Á. Borja , G. Chust , et al. 2016. “A Dark Hole in Our Understanding of Marine Ecosystems and Their Services: Perspectives From the Mesopelagic Community.” Frontiers in Marine Science 3: 31. 10.3389/fmars.2016.00031.

[eva70188-bib-0134] Staby, A. , A. Røstad , and S. Kaartvedt . 2011. “Long‐Term Acoustical Observations of the Mesopelagic Fish *Maurolicus muelleri* Reveal Novel and Varied Vertical Migration Patterns.” Marine Ecology Progress Series 441: 241–255. 10.3354/meps09363.

[eva70188-bib-0135] Standal, D. , and E. Grimaldo . 2021. “Lost in Translation? Practical‐ and Scientific Input to the Mesopelagic Fisheries Discourse.” Marine Policy 134: 104785. 10.1016/j.marpol.2021.104785.

[eva70188-bib-0136] Stukel, M. R. , J. P. Irving , T. B. Kelly , M. D. Ohman , C. K. Fender , and N. Yingling . 2023. “Carbon Sequestration by Multiple Biological Pump Pathways in a Coastal Upwelling Biome.” Nature Communications 14, no. 1: 2024. 10.1038/s41467-023-37771-8. PMC10090055 37041189

[eva70188-bib-0137] Suneetha, K. B. , and G. Nævdal . 2001. “Genetic and Morphological Stock Structure of the Pearlside, *Maurolicus muelleri* (Pisces, Sternoptychidae), Among Norwegian Fjords and Offshore Area.” Sarsia 86, no. 3: 191–201. 10.1080/00364827.2001.10420475.

[eva70188-bib-0138] Suneetha, K. B. , and A. G. V. Salvanes . 2001. “Population Genetic Structure of the Glacier Lanternfish, *Benthosema glaciale* (Myctophidae) in Norwegian Waters.” Sarsia 86: 203–212. 10.1080/00364827.2001.10420476.

[eva70188-bib-0139] Tacon, A. G. J. , and M. Metian . 2008. “Global Overview on the Use of Fish Meal and Fish Oil in Industrially Compounded Aquafeeds: Trends and Future Prospects.” Aquaculture 285, no. 1: 146–158. 10.1016/j.aquaculture.2008.08.015.

[eva70188-bib-0140] Team, R. C. 2025. R: A Language and Environment for Statistical Computing. R Foundation for Statistical Computing. https://www.R‐project.org/.

[eva70188-bib-0141] Tine, M. , H. Kuhl , P.‐A. Gagnaire , et al. 2014. “European Sea Bass Genome and Its Variation Provide Insights Into Adaptation to Euryhalinity and Speciation.” Nature Communications 5: 5770. 10.1038/ncomms6770. PMC4284805 25534655

[eva70188-bib-0142] Torgersen, T. , and S. Kaartvedt . 2001. “In Situ Swimming Behaviour of Individual Mesopelagic Fish Studied by Split‐Beam Echo Target Tracking.” ICES Journal of Marine Science 58, no. 1: 346–354. 10.1006/jmsc.2000.1016.

[eva70188-bib-0143] Waples, R. S. , A. E. Punt , and J. M. Cope . 2008. “Integrating Genetic Data Into Management of Marine Resources: How Can We Do It Better?” Fish and Fisheries 9, no. 4: 423–449. 10.1111/j.1467-2979.2008.00303.x.

[eva70188-bib-0144] Watkins, A. , S. Torkhildsen , and G. Nævdal . 1996. Allozyme Variation Within Three Species of Mesopelagic Fishes. Vol. 4, 1–22. Institutt for Fiskeri‐ og Marinbiologi. Rapport.

[eva70188-bib-0145] Weir, B. S. , and C. Cockerham . 1984. “Estimating F‐Statistics for the Analysis of Population Structure.” Evolution 38, no. 6: 1358–1370. 10.2307/2408641. 28563791

[eva70188-bib-0146] Westgaard, J.‐I. , and S.‐E. Fevolden . 2007. “Atlantic Cod ( *Gadus morhua* L.) in Inner and Outer Coastal Zones of Northern Norway Display Divergent Genetic Signature at Non‐Neutral Loci.” Fisheries Research 85: 306–315. 10.1016/j.fishres.2007.04.001.

[eva70188-bib-0147] Wickham, H. 2016. ggplot2: Elegant Graphics for Data Analysis. Springer‐Verlag New York.

[eva70188-bib-0148] Wright, S. 1943. “Isolation by Distance.” Genetics 28, no. 2: 114–138. 10.1093/genetics/28.2.114. 17247074 PMC1209196

[eva70188-bib-0149] Zane, L. , S. Marcato , L. Bargelloni , et al. 2006. “Demographic History and Population Structure of the Antarctic Silverfish *Pleuragramma antarcticum* .” Molecular Ecology 15, no. 14: 4499–4511. 10.1111/j.1365-294X.2006.03105.x. 17107479

