# Supplementary material for: Genetics in the Ocean's Twilight Zone: Population Structure of the Silvery Lightfish Across Its Distribution Range
Source: Evol Appl. 2026 Mar 23;19(3):e70188. doi: 10.1111/eva.70188 (PMC13093611; doi:10.1111/eva.70188)
Supplement: Supplementary file 1 — Table S1: List of the 30 discarded SNPs together with the reasons for them to be removed. Table S2: Compilation of pairwise F ST matrices from total non‐filtered data till the 170 individually genotyped SNPs retained for analyses: Table S3: Genetic differentiation between geographically explicit samples assessed with 170 SNPs: Heatmap of pairwise F ST values in the bottom diagonal and corresponding p‐values after 10,000 permutations in the top diagonal, with the ones significantly different from zero after FDR correction highlighted in boldface type. Table S4: Summary of information for loci involved in allele frequency clines. Table S5: Summary of Mantel and Partial Mantel tests calculated conducting genetic distance (F ST). Figure S1: Maurolicus muelleri : SNP mining using four samples in three different habitats. Figure S2: Proportion of polymorphic loci per sample using different sets of SNPs during the process of filtering of pool genome data as well as for the individually genotyped individuals using the Sequenom. Figure S3: Genotype accumulation curve calculated for the set of 170 polymorphic SNP loci using the total 863 individuals. Figure S4: A posteriori analysis of STRUCTURE outcome for the set of 170 loci following Puechmaille and Evanno's statistics, respectively. Figure S5: Barplot representing the proportion of individuals' ancestry to cluster at K3 to K5 after Bayesian clustering in STRUCTURE using the 170 total loci. Figure S6: Examples of cline latitudinal patterns for STRUCTURE Q‐score (a) and locus P105 (b). Figure S7: Detailed map of the studied area in the Greek Seas (a) and temperature at depth measured with CTD at the corresponding sampling sites (b). [file EVA-19-e70188-s001.docx]

**SUPPLEMENT**

**Genetics in the ocean’s twilight zone: Population structure of the silvery lightfish across its distribution range**

**TABLES**

**Table S1.-** List of the 30 discarded SNPs together with the reasons for them to be removed.

| **Locus** | **Reasons to remove** |
| --- | --- |
| P325 | 100% missing loci overall |
| P378 | 100% missing loci overall |
| P437 | 100% missing loci overall |
| P537 | 100% missing loci overall |
| P614 | 100% missing loci overall |
| P688 | 100% missing loci overall |
| P834 | 100% missing loci overall |
| P1153 | 100% missing loci overall |
| P1284 | 100% missing loci overall |
| P1295 | 100% missing loci overall |
| P1423 | 100% missing loci overall |
| P1479 | 100% missing loci overall |
| P1638 | 100% missing loci overall |
| P1675 | 100% missing loci overall |
| P1774 | 100% missing loci overall |
| P1775 | 100% missing loci overall |
| P1869 | 100% missing loci overall |
| P1900 | 100% missing loci overall |
| P1953 | 100% missing loci overall |
| P660 | 66.3% missing loci overall |
| P974 | 70.2% missing loci overall |
| P1784 | 81.35% missing loci overall |
| P1246 | Empty in various samples |
| P1251 | Empty in various samples |
| P1315 | Empty in various samples |
| P1346 | Empty in various samples |
| P1520 | Empty in various samples |
| P1568 | Empty in various samples |
| P1832 | Empty in various samples |
| P1922 | Empty in various samples |

**Table S2.-** Compilation of pairwise *F*_ST_ matrices from total non-filtered data till the 170 individually genotyped SNPs retained for analyses:

1. Pairwise *F*_ST_ from pool genome sequencing: 216,150 non-filtered SNPs (lower diagonal) and 12,000 filtered SNPs (upper diagonal).

|  | **Boknafjord** | **CelticSea** | **Atlantic_47N** | **IonianSea** |
| --- | --- | --- | --- | --- |
| **Boknafjord** |  | 0.014 | 0.044 | 0.260 |
| **CelticSea** | 0.025 |  | 0.000 | 0.216 |
| **Atlantic_47N** | 0.047 | 0.000 |  | 0.121 |
| **IonianSea** | 0.284 | 0.259 | 0.159 |  |

1. Pairwise *F*_ST_ at 170 SNPs: pool sequencing (lower diagonal) and individual genotyping (upper diagonal).

|  | **Boknafjord** | **CelticSea** | **Atlantic_47N** | **IonianSea** |
| --- | --- | --- | --- | --- |
| **Boknafjord** |  | 0.072 | 0.098 | 0.339 |
| **CelticSea** | 0.041 |  | 0.027 | 0.302 |
| **Atlantic_47N** | 0.055 | 0.000 |  | 0.199 |
| **IonianSea** | 0.283 | 0.260 | 0.172 |  |

**Table S3.-** Genetic differentiation between geographically explicit samples assessed with 170 SNPs: Heatmap of pairwise *F*_ST_ values in the bottom diagonal and corresponding *P*-values after 10,000 permutations in the top diagonal, with the ones significantly different from zero after FDR correction highlighted in boldface type. Greener colours indicate low differentiation increasing towards red to indicate larger differentiation. Sample names shaded in grey have sampling sizes ranging from 10 to 18 individuals.

|  | **Osterfj** | **Byfj** | **Korsfj** | **Bjørnafj** | **Boknafj** | **Vesterål** | **Norway63N** | **Iceland** | **Norway60N** | **Norway59N** | **AtlanticXIIc** | **Celtic** | **Flemish** | **Atlantic47N** | **BayBiscay** | **Cantabrian** | **Atlantic40N** | **Morocco** | **Alborán** | **NAegean** | **NEuboean** | **Ionian** | **Cretan** |
| --- | --- | --- | --- | --- | --- | --- | --- | --- | --- | --- | --- | --- | --- | --- | --- | --- | --- | --- | --- | --- | --- | --- | --- |
| **Osterfj** | * | 0.855 | 0.830 | 0.752 | **0.002** | **0.030** | **0.000** | **0.000** | **0.000** | **0.000** | **0.000** | **0.000** | **0.000** | **0.000** | **0.000** | **0.000** | **0.000** | **0.000** | **0.000** | **0.000** | **0.000** | **0.000** | **0.000** |
| **Byfj** | 0.000 | * | 0.882 | 0.979 | 0.981 | 0.141 | **0.000** | **0.000** | **0.000** | **0.000** | **0.000** | **0.000** | **0.000** | **0.000** | **0.000** | **0.000** | **0.000** | **0.000** | **0.000** | **0.000** | **0.000** | **0.000** | **0.000** |
| **Korsfj** | 0.000 | 0.000 | * | 0.879 | 0.491 | 0.139 | **0.000** | **0.000** | **0.000** | **0.000** | **0.000** | **0.000** | **0.000** | **0.000** | **0.000** | **0.000** | **0.000** | **0.000** | **0.000** | **0.000** | **0.000** | **0.000** | **0.000** |
| **Bjørnafj** | 0.000 | 0.000 | 0.000 | * | 0.571 | **0.013** | **0.000** | **0.000** | **0.000** | **0.000** | **0.000** | **0.000** | **0.000** | **0.000** | **0.000** | **0.000** | **0.000** | **0.000** | **0.000** | **0.000** | **0.000** | **0.000** | **0.000** |
| **Boknafj** | 0.007 | 0.000 | 0.000 | 0.000 | * | **0.004** | **0.000** | **0.000** | **0.000** | **0.000** | **0.000** | **0.000** | **0.000** | **0.000** | **0.000** | **0.000** | **0.000** | **0.000** | **0.000** | **0.000** | **0.000** | **0.000** | **0.000** |
| **Vesterål** | 0.009 | 0.005 | 0.005 | 0.010 | 0.012 | * | **0.000** | **0.000** | **0.000** | **0.000** | **0.000** | **0.000** | **0.001** | **0.000** | **0.000** | **0.000** | **0.000** | **0.000** | **0.000** | **0.000** | **0.000** | **0.000** | **0.000** |
| **Norway63N** | 0.086 | 0.096 | 0.081 | 0.081 | 0.096 | 0.048 | * | 0.162 | 0.999 | 0.992 | 0.721 | 0.663 | 0.050 | **0.000** | **0.000** | **0.000** | **0.000** | **0.000** | **0.000** | **0.000** | **0.000** | **0.000** | **0.000** |
| **Iceland** | 0.104 | 0.117 | 0.102 | 0.102 | 0.121 | 0.065 | 0.012 | * | 0.519 | 0.175 | 0.552 | 0.402 | 0.594 | **0.000** | **0.000** | **0.000** | **0.000** | **0.000** | **0.000** | **0.000** | **0.000** | **0.000** | **0.000** |
| **Norway60N** | 0.089 | 0.091 | 0.082 | 0.088 | 0.102 | 0.054 | 0.000 | 0.000 | * | 1.000 | 0.821 | **0.026** | 0.345 | **0.000** | **0.000** | **0.000** | **0.000** | **0.000** | **0.000** | **0.000** | **0.000** | **0.000** | **0.000** |
| **Norway59N** | 0.083 | 0.086 | 0.077 | 0.082 | 0.096 | 0.042 | 0.000 | 0.005 | 0.000 | * | 0.997 | 0.959 | **0.008** | **0.000** | **0.000** | **0.000** | **0.000** | **0.000** | **0.000** | **0.000** | **0.000** | **0.000** | **0.000** |
| **AtlanticXIIc** | 0.094 | 0.096 | 0.089 | 0.094 | 0.108 | 0.054 | 0.000 | 0.000 | 0.000 | 0.000 | * | 0.251 | 0.075 | **0.000** | **0.000** | **0.000** | **0.000** | **0.000** | **0.000** | **0.000** | **0.000** | **0.000** | **0.000** |
| **Celtic** | 0.079 | 0.082 | 0.075 | 0.080 | 0.093 | 0.046 | 0.000 | 0.001 | 0.004 | 0.000 | 0.001 | * | 0.316 | **0.000** | **0.000** | **0.000** | **0.000** | **0.000** | **0.000** | **0.000** | **0.000** | **0.000** | **0.000** |
| **Flemish** | 0.087 | 0.090 | 0.082 | 0.080 | 0.103 | 0.049 | 0.019 | 0.000 | 0.002 | 0.013 | 0.007 | 0.002 | * | **0.000** | **0.000** | **0.000** | **0.000** | **0.000** | **0.000** | **0.000** | **0.000** | **0.000** | **0.000** |
| **Atlantic47N** | 0.109 | 0.106 | 0.103 | 0.106 | 0.114 | 0.074 | 0.041 | 0.052 | 0.046 | 0.045 | 0.049 | 0.031 | 0.055 | * | 0.387 | **0.000** | 0.454 | **0.000** | **0.000** | **0.000** | **0.000** | **0.000** | **0.000** |
| **Bay_Biscay** | 0.122 | 0.112 | 0.117 | 0.119 | 0.122 | 0.088 | 0.066 | 0.078 | 0.078 | 0.080 | 0.082 | 0.056 | 0.090 | 0.000 | * | **0.002** | 0.996 | **0.000** | **0.000** | **0.000** | **0.000** | **0.000** | **0.000** |
| **Cantabrian** | 0.176 | 0.172 | 0.169 | 0.169 | 0.176 | 0.147 | 0.123 | 0.130 | 0.129 | 0.131 | 0.132 | 0.104 | 0.139 | 0.025 | 0.011 | * | **0.005** | **0.021** | **0.000** | **0.000** | **0.000** | **0.000** | **0.000** |
| **Atlantic40N** | 0.122 | 0.115 | 0.116 | 0.120 | 0.126 | 0.093 | 0.067 | 0.075 | 0.076 | 0.072 | 0.080 | 0.056 | 0.080 | 0.000 | 0.000 | 0.009 | * | **0.000** | **0.000** | **0.000** | **0.000** | **0.000** | **0.000** |
| **Morocco** | 0.213 | 0.206 | 0.207 | 0.209 | 0.214 | 0.185 | 0.174 | 0.183 | 0.178 | 0.178 | 0.182 | 0.150 | 0.184 | 0.051 | 0.029 | 0.005 | 0.025 | * | **0.000** | **0.000** | **0.000** | **0.000** | **0.000** |
| **Alborán** | 0.338 | 0.348 | 0.334 | 0.331 | 0.342 | 0.339 | 0.363 | 0.370 | 0.322 | 0.334 | 0.327 | 0.286 | 0.372 | 0.162 | 0.128 | 0.080 | 0.115 | 0.043 | * | **0.000** | **0.000** | **0.000** | **0.000** |
| **NAegean** | 0.360 | 0.357 | 0.352 | 0.347 | 0.354 | 0.355 | 0.370 | 0.391 | 0.349 | 0.358 | 0.355 | 0.326 | 0.390 | 0.212 | 0.177 | 0.125 | 0.168 | 0.091 | 0.062 | * | **0.000** | **0.000** | 0.516 |
| **NEuboean** | 0.214 | 0.214 | 0.206 | 0.208 | 0.214 | 0.191 | 0.198 | 0.213 | 0.194 | 0.195 | 0.199 | 0.177 | 0.209 | 0.108 | 0.093 | 0.072 | 0.087 | 0.084 | 0.160 | 0.174 | * | **0.000** | **0.000** |
| **Ionian** | 0.327 | 0.323 | 0.321 | 0.318 | 0.323 | 0.319 | 0.337 | 0.355 | 0.321 | 0.330 | 0.326 | 0.295 | 0.353 | 0.181 | 0.146 | 0.110 | 0.144 | 0.074 | 0.063 | 0.049 | 0.133 | * | **0.000** |
| **Cretan** | 0.365 | 0.364 | 0.357 | 0.351 | 0.362 | 0.367 | 0.382 | 0.402 | 0.352 | 0.360 | 0.359 | 0.330 | 0.401 | 0.215 | 0.185 | 0.129 | 0.171 | 0.097 | 0.075 | 0.000 | 0.179 | 0.062 | * |

**Table S4.-** Summary of information for loci involved in allele frequency clines. Loadings on the first axis of the DAPC with values highlighted in boldface font corresponding to the loci that contributed the most to the genetic differentiation. The cline model represents the best-fitting model for each locus, as determined by the sample-size–corrected Akaike Information Criterion (AICc), selected from among the 15 models tested using the HZAR software. Cline centre in km, with loci shaded in grey corresponding to the ones that fit within the limits of the reference cline built with STRUCTURE-Q score in a geographic transect of circa 8000 km extending from Vesterålen to the Cretan Sea. Gene predictions identified using BLAST on the SNP flanking regions.

| **Locus** | **DAPC** | **Cline model** | **Cline centre** | **BLAST** |
| --- | --- | --- | --- | --- |
| P1446 | **0.23** | fixR | 107 |  |
| P1691 | **0.21** | typN | 2340 | PREDICTED: *Calypte anna* ADAM metallopeptidase domain 12 (ADAM12), transcript variant X1 |
| P635 | 0.18 | typL | 2841 |  |
| P1636 | **0.27** | optR | 3035 |  |
| P1770 | **0.32** | fixR | 3064 |  |
| P1945 | **0.26** | fixR | 3157 |  |
| P709 | 0.14 | typR | 3160 |  |
| P105 | **0.24** | optB | 3162 | *Paramormyrops kingsleyae* disintegrin and metalloproteinase domain-containing protein 12-like |
| P1267 | **0.24** | optB | 3176 |  |
| P1569 | 0.14 | typR | 3183 |  |
| P942 | 0.19 | optM | 3230 | PREDICTED: *Sparus aurata* zinc finger and BTB domain containing 39 (zbtb39) |
| P1458 | **0.23** | typR | 3251 |  |
| P676 | **0.33** | optN | 3482 |  |
| P1989 | **0.23** | optN | 3573 | PREDICTED: *Hypomesus transpacificus* zinc finger protein 609-like (LOC124481548), transcript variant X2 |
| P402 | **0.32** | typR | 3588 |  |
| P1500 | 0.20 | typN | 3597 |  |
| P1856 | **0.23** | typN | 3635 |  |
| P1606 | 0.09 | fixR | 3815 |  |
| P989 | 0.17 | typR | 4191 | PREDICTED: *Seriola dumerili* MAX interactor 1, dimerization protein (mxi1), transcript variant X2 |
| P1668 | 0.06 | optN | 3155 |  |
| P253 | 0.10 | fixN | 3338 | PREDICTED: *Salvelinus fontinalis* anoctamin 5a (ano5a), transcript variant X2 |
| P359 | 0.18 | fixR | 3468 |  |
| P318 | 0.12 | fixN | 41 |  |
| P1337 | 0.01 | typM | 396 |  |
| P481 | 0.14 | fixN | 1617 |  |
| P790 | 0.20 | fixN | 1701 |  |
| P1345 | 0.05 | fixN | 2348 |  |
| P1661 | **0.24** | optN | 2634 |  |
| P1133 | 0.13 | optN | 2670 |  |
| P1010 | 0.05 | fixN | 2732 |  |
| P449 | 0.03 | fixN | 2743 |  |
| P267 | 0.02 | optN | 2778 |  |
| P045 | 0.16 | fixR | 2778 |  |
| P1863 | **0.21** | fixR | 2782 |  |
| P102 | 0.02 | optN | 3036 |  |
| P296 | 0.06 | optN | 3053 |  |
| P1883 | 0.03 | fixN | 3144 |  |
| P1682 | 0.01 | fixN | 3170 |  |
| P1833 | 0.11 | fixN | 3342 |  |
| P1241 | 0.07 | fixN | 3376 |  |
| P661 | 0.02 | fixN | 3395 |  |
| P303 | 0.20 | fixN | 3403 |  |
| P356 | 0.05 | fixN | 3417 |  |
| P1930 | 0.00 | fixN | 3452 |  |
| P307 | 0.20 | optN | 3477 |  |
| P793 | 0.04 | fixN | 3625 |  |
| P1981 | 0.13 | fixN | 3631 |  |
| P1757 | 0.05 | fixN | 3654 |  |
| P643 | 0.08 | fixN | 3686 |  |
| P479 | 0.04 | fixN | 3762 |  |
| P545 | 0.03 | fixN | 3784 |  |
| P488 | 0.11 | fixN | 3971 |  |
| P002 | 0.16 | optN | 4291 |  |
| P584 | 0.05 | optN | 4302 |  |
| P1062 | **0.25** | optN | 4546 |  |
| P654 | 0.04 | fixN | 4656 |  |
| P1768 | **0.22** | fixN | 4682 |  |
| P003 | 0.11 | fixN | 4937 |  |
| P1929 | 0.18 | fixN | 5236 |  |
| P1516 | 0.05 | fixN | 5921 |  |
| P015 | 0.15 | fixN | 6662 |  |
| P1798 | 0.02 | typL | 7379 |  |
| P1464 | 0.09 | fixN | 5553 |  |
| P1732 | 0.08 | fixN | 1920 |  |
| P041 | 0.14 | fixN | 2612 |  |
| P789 | **0.25** | optN | 2807 |  |
| P1714 | 0.03 | optB | 2880 |  |
| P1855 | 0.15 | optN | 2971 |  |
| P1222 | 0.02 | fixN | 5647 |  |
| P414 | 0.13 | optN | 6309 |  |
| P550 | 0.06 | NullModel | na |  |
| P443 | 0.16 | optN | 2596 |  |

**Table S5.-** Summary of Mantel and Partial Mantel tests calculated conducting genetic distance (*F*_ST_). Geographic distance (Geo) was calculated as the shortest water distance and temperature distance (Temp) refers to the average temperature measured in summer between 2005 and 2012 at 200 m depth. Partial Mantel tests are corrected by controlling for the effect of the matrix indicated in brackets. Boldface font depicts statistically significant *P*-values.

| **Test** | **Matrices** | **r_xy_** | ***P*-value** |
| --- | --- | --- | --- |
| **Mantel Test** | Geo_*F*_ST_ | 0.784 | **0.0001** |
|  | Temp_*F*_ST_ | 0.712 | **0.0001** |
|  | Geo_Temp | 0.790 | **0.0001** |
|  |  |  |  |
| **Partial Mantel test** | Geo_*F*_ST_(Temp) | 0.514 | **0.0005** |
|  | Temp_*F*_ST_(Geo) | 0.243 | **0.0064** |

**FIGURES**

| 1. PCA 216,150 non-filtered SNPs   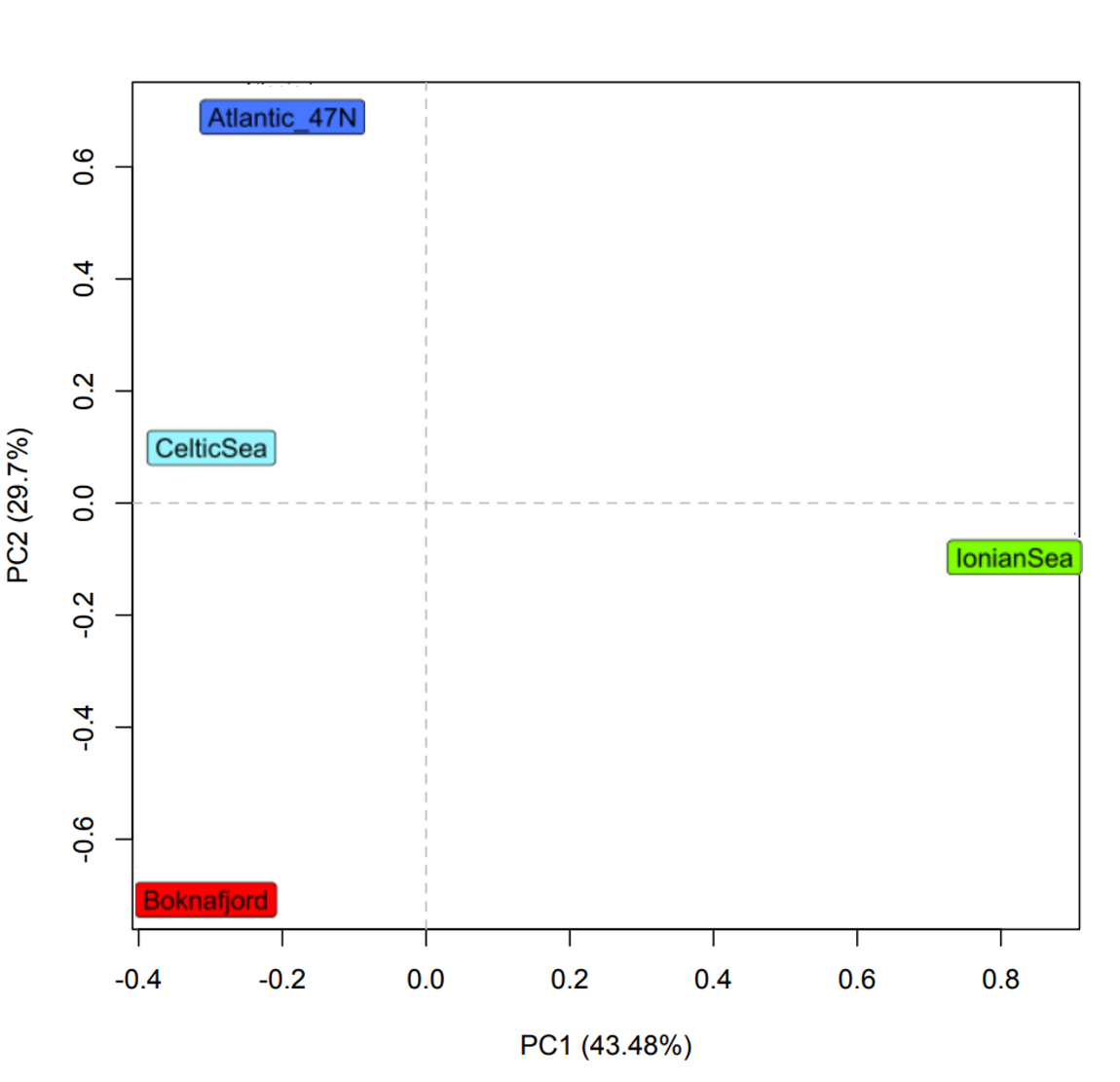 |
| --- |
| 1. PCA 12000 filtered SNPs    |

| 1. PCA 170 SNPs (individually genotyped) |
| --- |

**Fig. S1.** *Maurolicus muelleri***:** SNP mining using four samples in three different habitats. Principal Component Analysis built with the total 216,150 non-filtered SNPs obtained from pool sequencing (a) and with the 12,000 filtered SNP and c) with the array of 170 loci retained for statistical analysis individual genotyped.

**Fig. S2.** Proportion of polymorphic loci per sample using different sets of SNPs during the process of filtering of pool genome data as well as for the individually genotyped individuals using the Sequenom. Sampling sizes per location are N=10 for pool genome sequencing and N=49 (Boknafjord), N=38 (Celtic Sea), N=41 (Atlantic_47N) and N=47 (Ionian Sea) for individual genotyping (Sequenom), respectively.

**
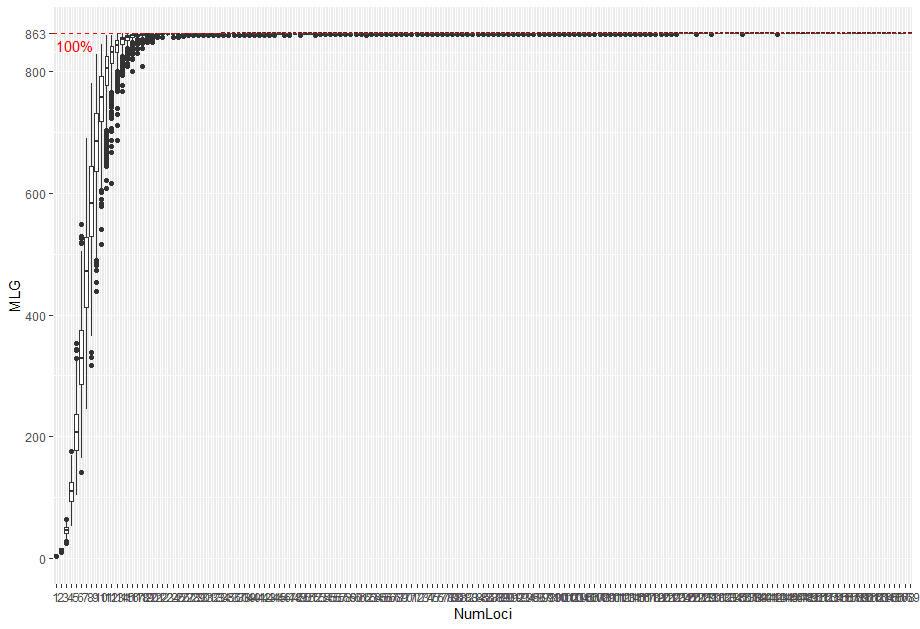
**

**Fig. S3.** Genotype accumulation curve calculated for the set of 170 polymorphic SNP loci using the total 863 individuals.

**Fig. S4.** *A posteriori* analysis of STRUCTURE outcome for the set of 170 loci following Puechmaille and Evanno’s statistics, respectively.

| a)  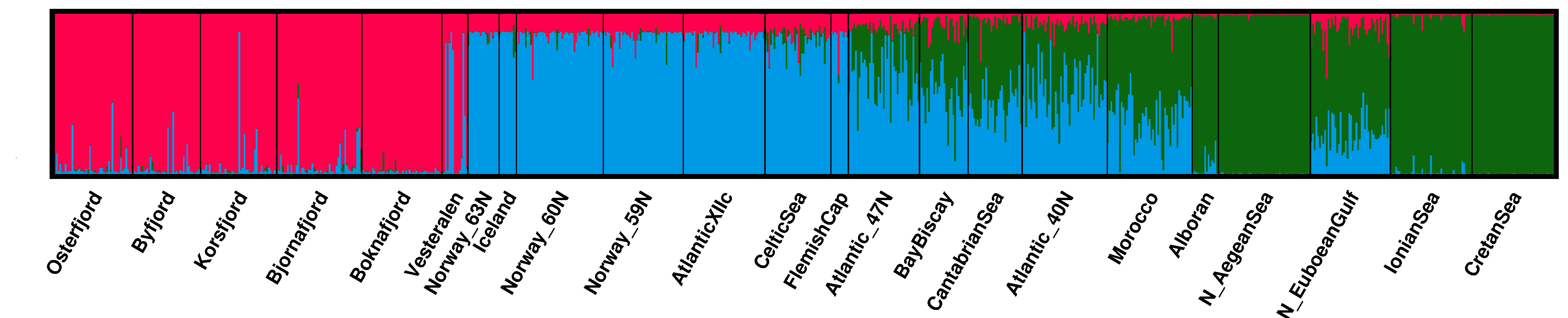 |
| --- |
| b)  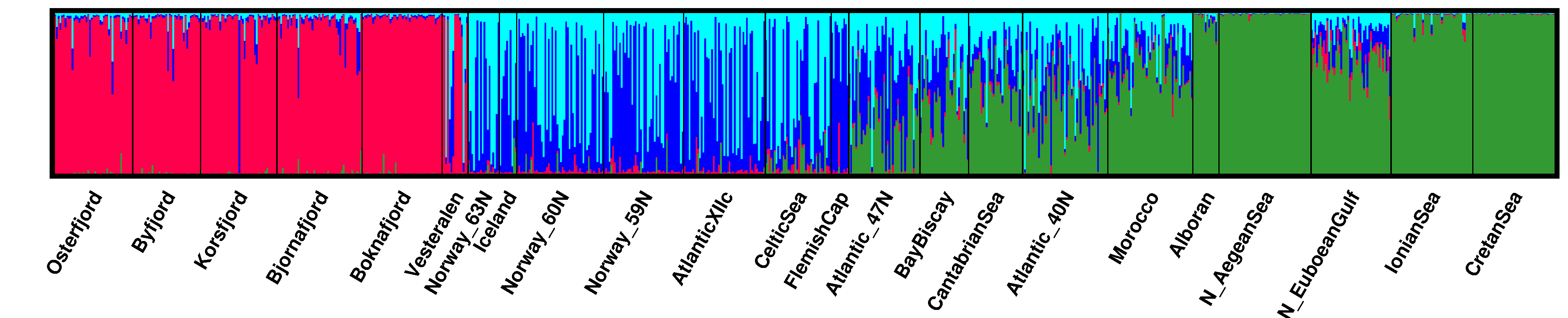 |
| c) |


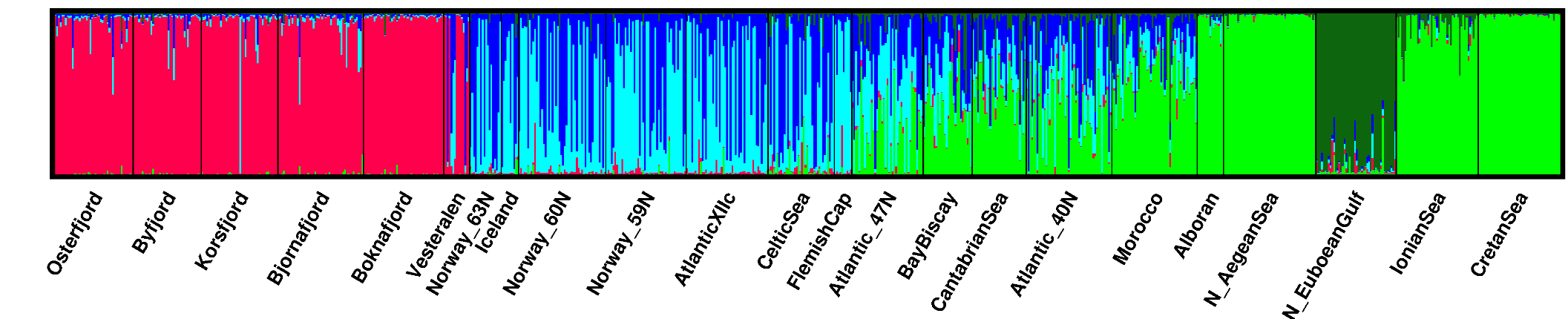


**Fig. S5.** Barplot representing the proportion of individuals’ ancestry to cluster at K3 to K5 after Bayesian clustering in STRUCTURE using the 170 total loci.

| a)  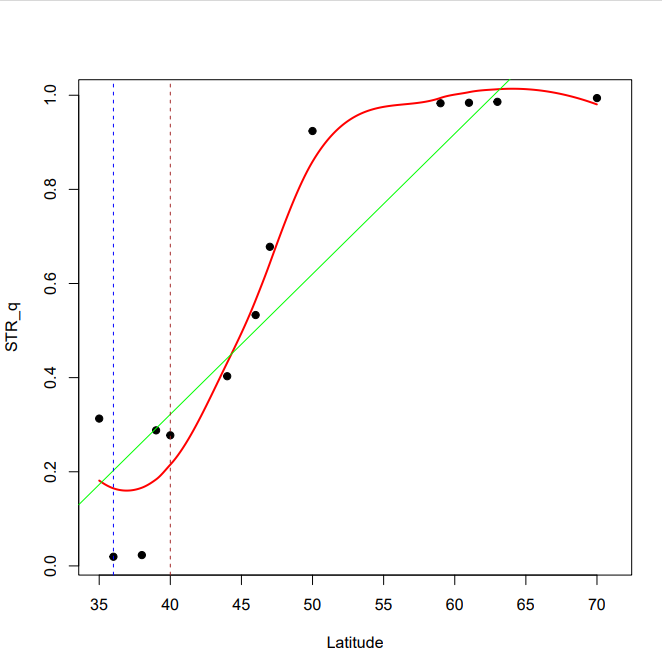 |
| --- |
| b)  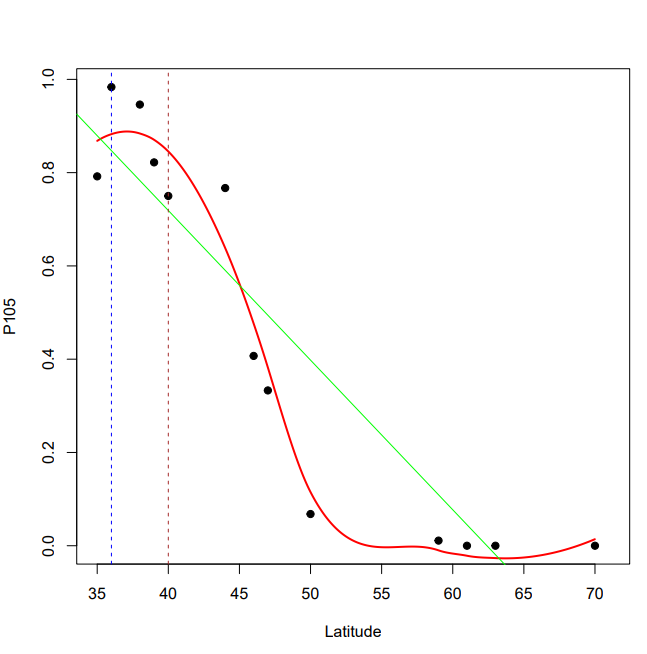 |

**Fig. S6.** Examples of cline latitudinal patterns for STRUCTURE Q-score (a) and locus P105 (b).

| a)  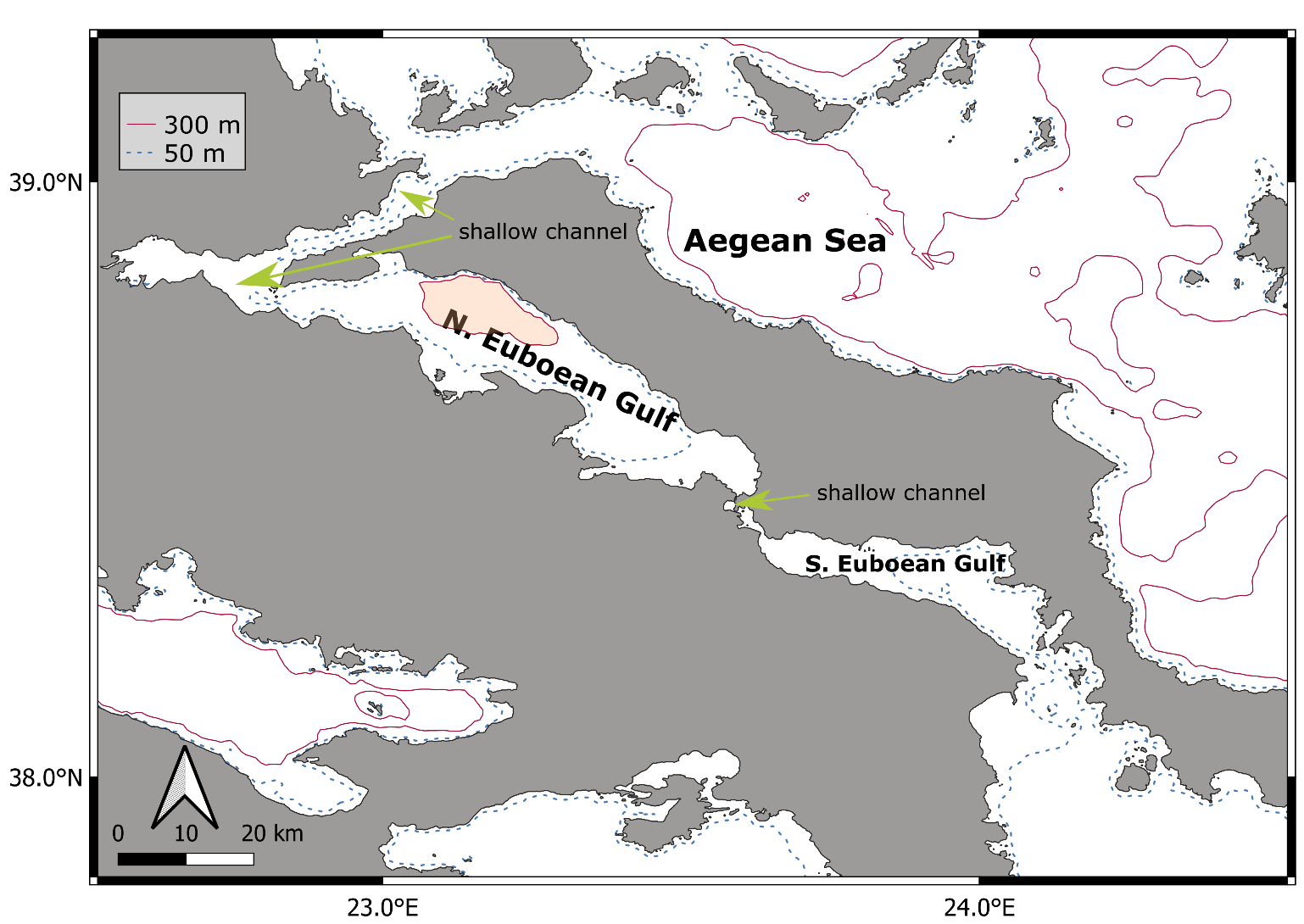 |
| --- |
| b)  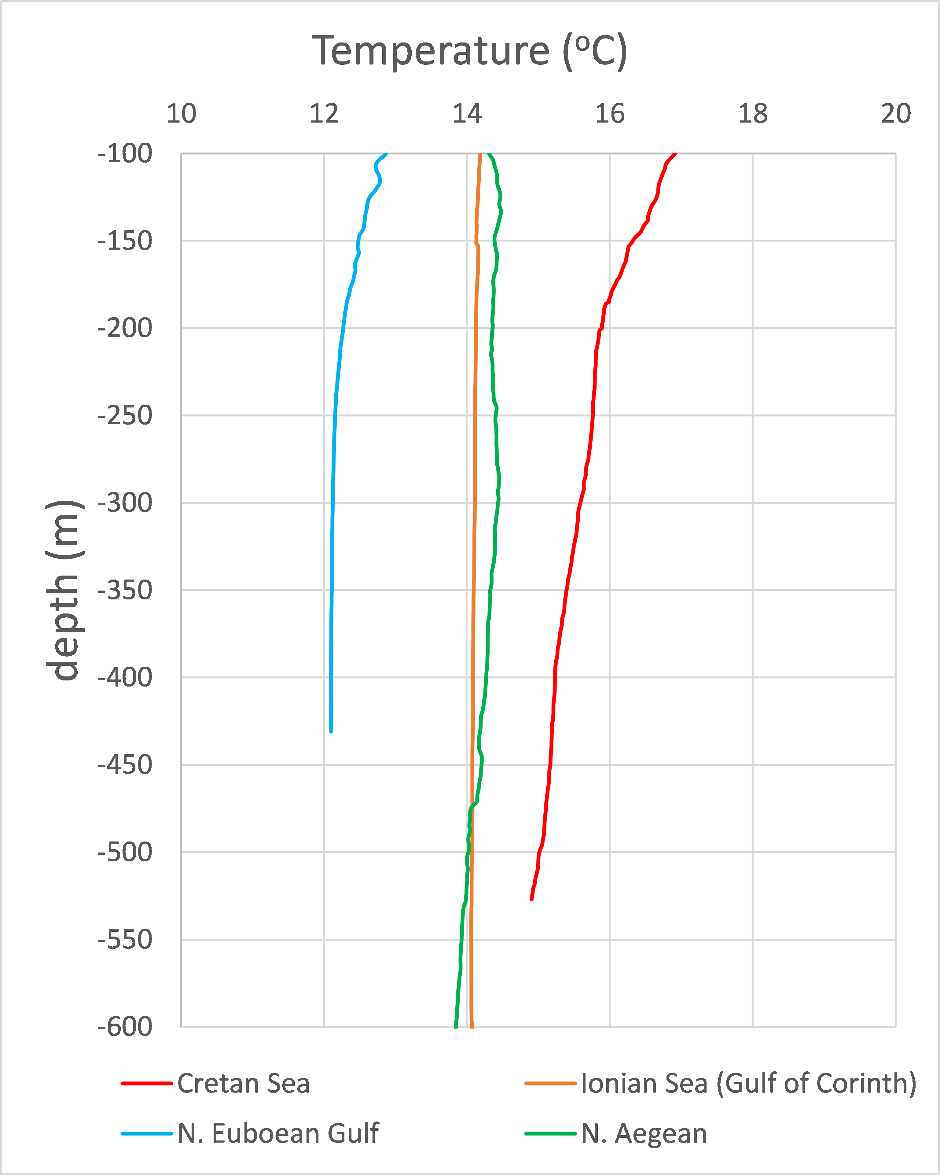 |

**Fig S7.** Detailed map of the studied area in the Greek Seas (a) and temperature at depth measured with CTD at the corresponding sampling sites (b).
